# Supplementary material for: Crowdsourced mapping of unexplored target space of kinase inhibitors
Source: Nat Commun. 2021 Jun 3;12:3307. doi: 10.1038/s41467-021-23165-1 (PMC8175708; doi:10.1038/s41467-021-23165-1)
Supplement: Supplementary file 1 — Supplementary Information [file 41467_2021_23165_MOESM1_ESM.pdf]

Supplementary Information

Crowdsourced mapping of unexplored target space of kinase inhibitors

Anna Cichońska[1,2,3]*, Balaguru Ravikumar[1]*, Robert J Allaway[4]*, Fangping Wan[5], Sungjoon Park[6], Olexandr Isayev[7], Shuya Li[5], Michael Mason[4], Andrew Lamb[4], Ziaurrehman Tanoli[1], Minji Jeon[6], Sunkyu Kim[6], Mariya Popova[7], Stephen Capuzzi[8], Jianyang Zeng[5], Kristen Dang[4], Gregory Koytiger[9], Jaewoo Kang[6], Carrow I. Wells[10], Timothy M. Willson[10], **The IDG-DREAM Drug-Kinase Binding Prediction Challenge Consortium**, Tudor I. Oprea[11], Avner Schlessinger[12], David H. Drewry[10], Gustavo Stolovitzky[13], Krister Wennerberg[14]**, Justin Guinney[4]**, Tero Aittokallio[1,2,15,16,17]**

*These authors contributed equally

**These authors jointly supervised this work

[1]Institute for Molecular Medicine Finland (FIMM), University of Helsinki, Helsinki, Finland
[2]Department of Computer Science, Helsinki Institute for Information Technology (HIIT), Aalto University, Espoo, Finland
[3]Department of Computing, University of Turku, Turku, Finland
[4]Computational Oncology, Sage Bionetworks, Seattle, WA, USA
[5]Institute for Interdisciplinary Information Sciences, Tsinghua University, Beijing, China, 100084
[6]Department of Computer Science and Engineering, Korea University, Seoul, Republic of Korea
[7]Department of Chemistry, Carnegie Mellon University, Pittsburgh, PA
[8]Laboratory for Molecular Modeling, Division of Chemical Biology and Medicinal Chemistry, UNC Eshelman School of Pharmacy, University of North Carolina, Chapel Hill, North Carolina 27599, United States
[9]Immuneering Corporation, Cambridge, MA, USA
[10]Structural Genomics Consortium, UNC Eshelman School of Pharmacy, University of North Carolina at Chapel Hill, Chapel Hill, North Carolina, USA
[11]Translational Informatics Division and Comprehensive Cancer Center, University of New Mexico School of Medicine, Albuquerque, New Mexico, USA
[12]Department of Pharmacological Sciences, Icahn School of Medicine at Mount Sinai, New York, New York, USA
[13]IBM T J Watson Research Center, IBM, Yorktown Heights, NY, USA
[14]Biotech Research and Innovation Centre (BRIC), University of Copenhagen, Copenhagen, Denmark
[15]Department of Mathematics and Statistics, University of Turku, Turku, Finland
[16]Institute for Cancer Research, Oslo University Hospital, Oslo, Norway
[17]Oslo Centre for Biostatistics and Epidemiology (OCBE), University of Oslo, Oslo, Norway

Correspondence should be addressed to: K.W. (krister.wennerberg@bric.ku.dk)., J.G. (justin.guinney@sagebionetworks.org) or T.A. (tero.aittokallio@helsinki.fi)

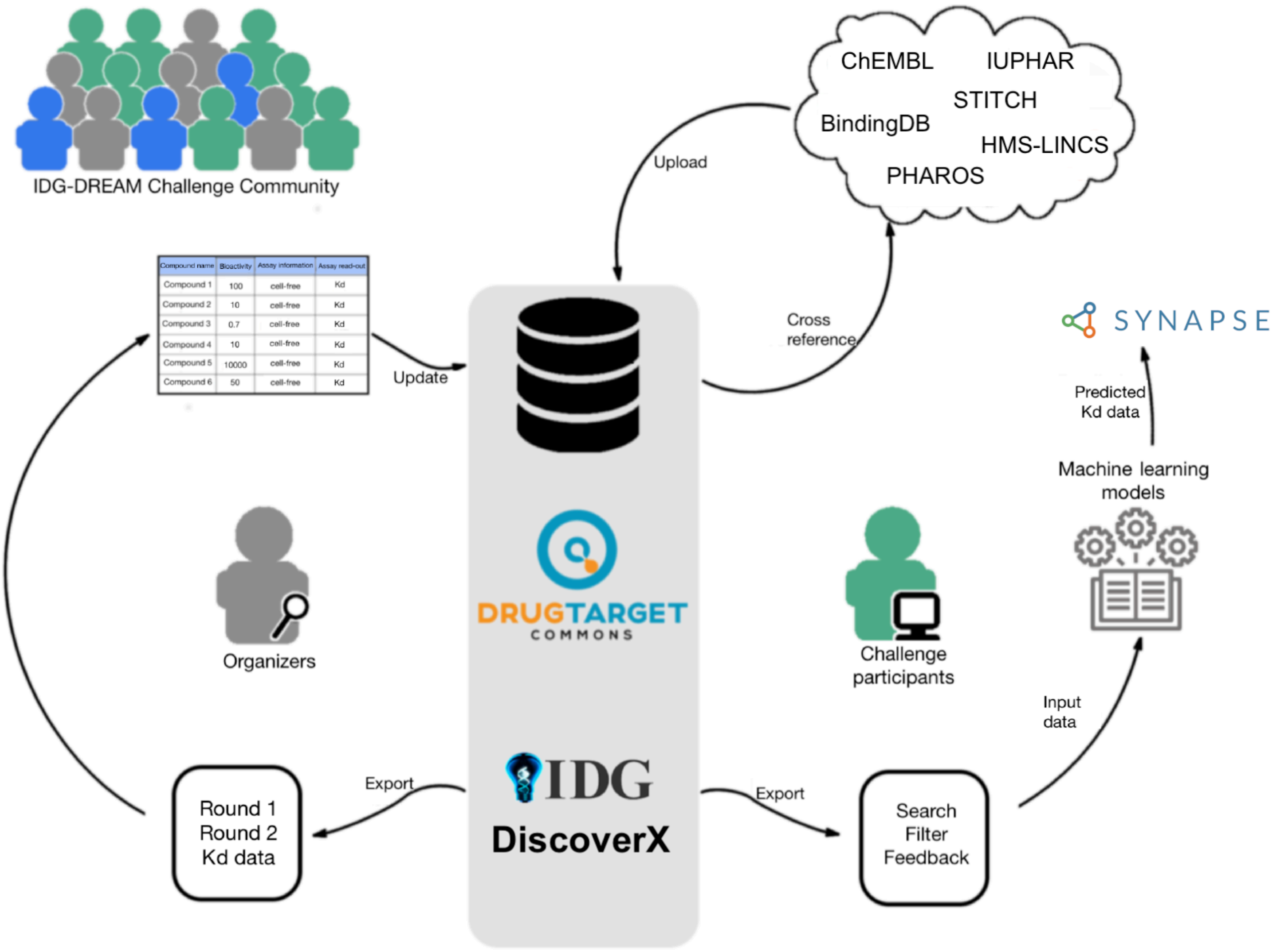

Supplementary Figure 1. The use of DrugTargetCommons (DTC) open-data platform in the Challenge. The test bioactivity data were provided by the Illuminating the Druggable Genome (IDG) program, and the multi-dose dissociation constant (K_d) data for Rounds 1 and 2 were generated by DiscoverX (Eurofins Corporation).

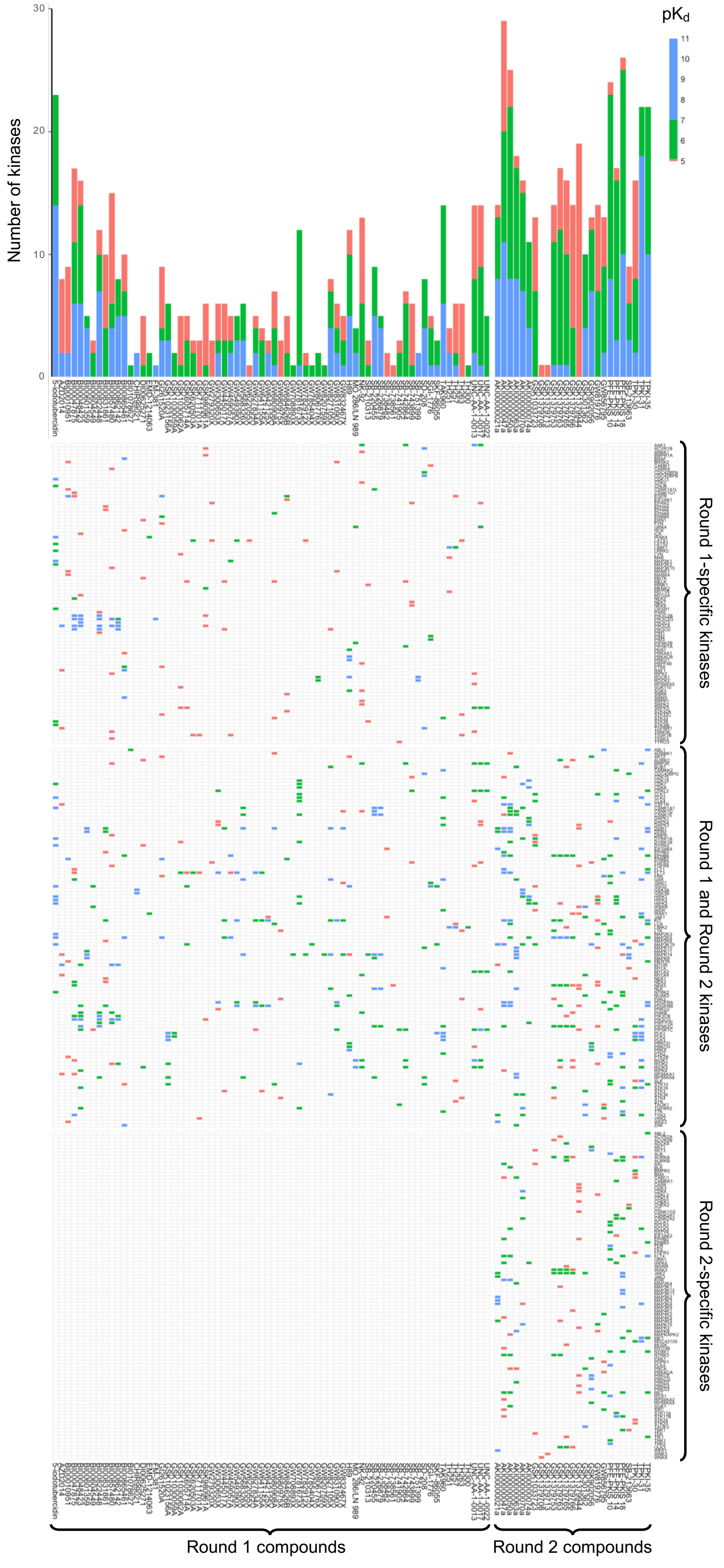

Supplementary Figure 2. Multi-dose dissociation constant (pK_d) heatmap and distributions of the compound-kinase activities used as the test data in the Challenge Rounds 1 and 2 (available in Supplementary Data 1). Source data are provided as a Source Data file (1).

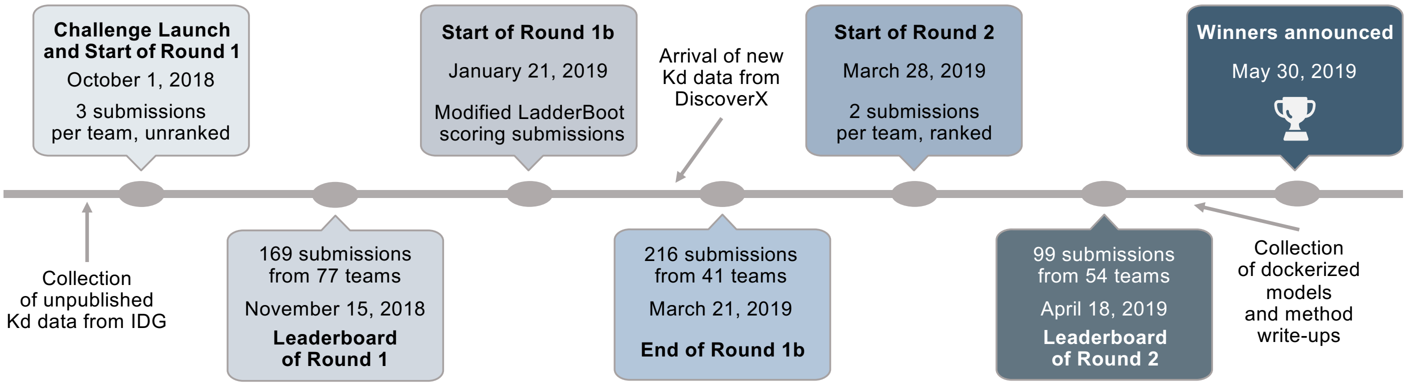

Supplementary Figure 3. Timeline of the IDG-DREAM Drug-Kinase Binding prediction Challenge. The ad-hoc leaderboard Round 1b was implemented while waiting for the new dissociation constant (K_d) bioactivity data from DiscoverX.

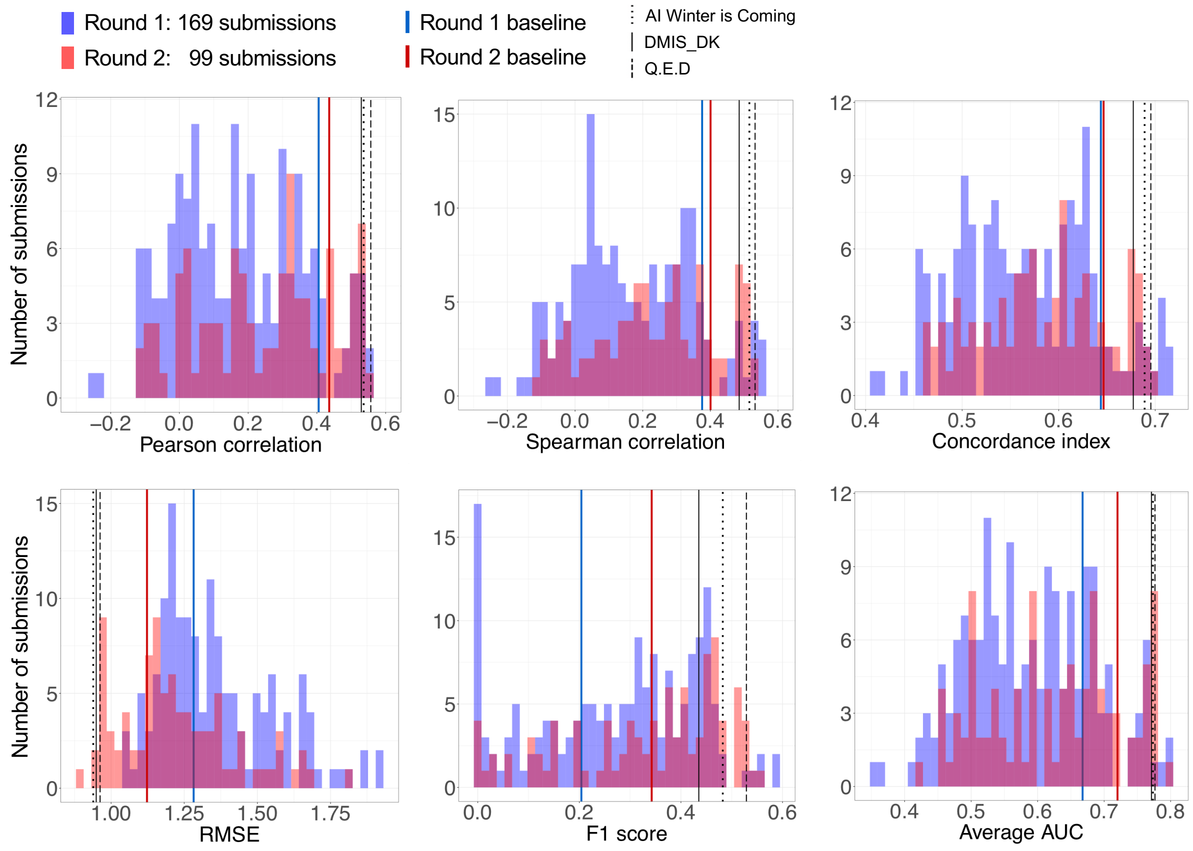

Supplementary Figure 4. Distributions of the Round 1 and Round 2 predictions, as evaluated with six scoring metrics and compared to the baseline model (thicker solid vertical lines) as well as top-performing teams in Round 2 (thinner solid, dotted and dashed vertical lines). 33 submissions with RMSE > 2 were omitted from the RMSE distribution. Abbreviations: RMSE, Root Mean Square Error; AUC, area under curve. Source data are provided as a Source Data file (1).

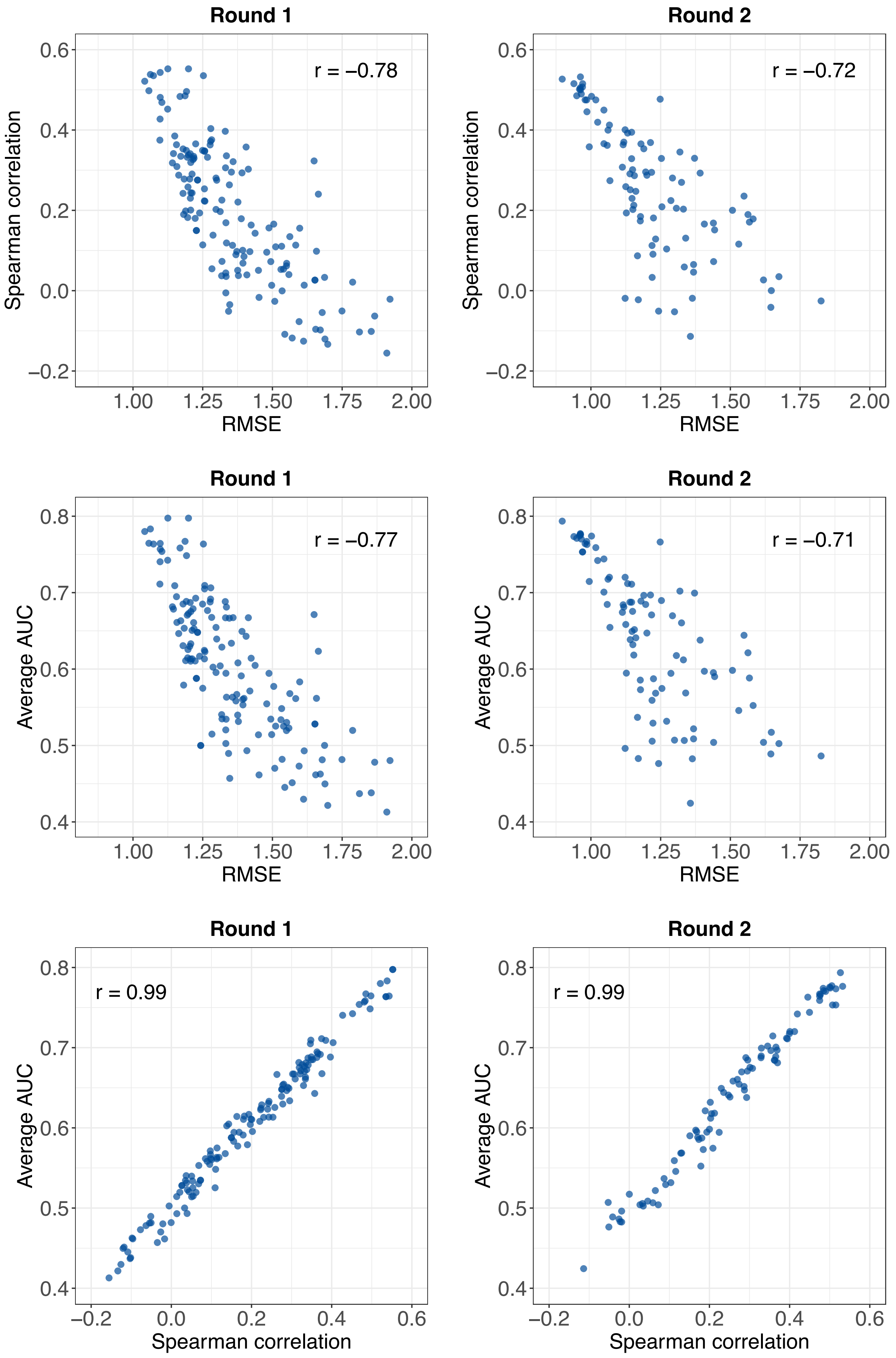

Supplementary Figure 5. Relationship between the two winning metrics (Spearman correlation and RMSE), and the tie-breaking metric (average AUC). Each point corresponds to one of the submissions in Round 1 (left panel) or in Round 2 (right panel); 33 submissions with RMSE > 2 were omitted. Abbreviations: RMSE, Root Mean Square Error; AUC, area under curve; r, Pearson correlation. Source data are provided as a Source Data file (1).

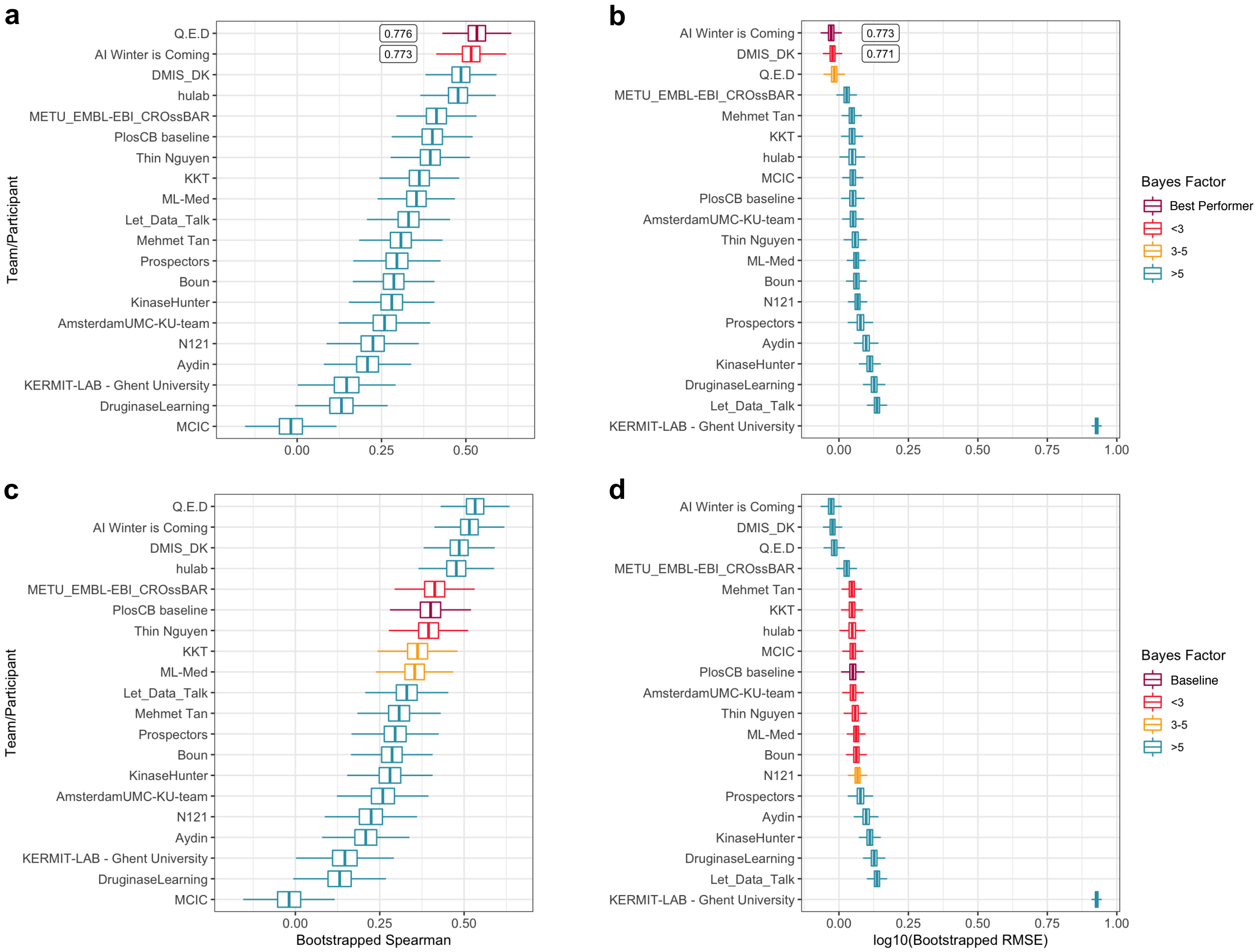

Supplementary Figure 6. Bayes factor analysis of the qualified participants/teams in Round 2. Upper panel: comparison against the top-performing model using (a) bootstrapped Spearman correlation and (b) bootstrapped RMSE. Lower panel: comparison against the baseline model using (c) bootstrapped Spearman correlation and (d) bootstrapped RMSE. Each boxplot summarizes the Spearman correlations or the RMSE and Bayes factors calculated from n=10000 bootstrapped predictions. In the boxplots, the vertical lines drawn in the middle denote the median, and the left and right hinges correspond to the 25th and 75th percentiles, respectively. The left and right whiskers denote the smallest and largest values, respectively, no further than 1.5 times the inter-quartile range. For visual clarity due to large sample size, the outliers (data points that are not included between the whiskers) are not displayed, but they are included in the Bayes factor calculations. Abbreviation: RMSE, Root Mean Square Error. Source data are provided as a Source Data file (1).

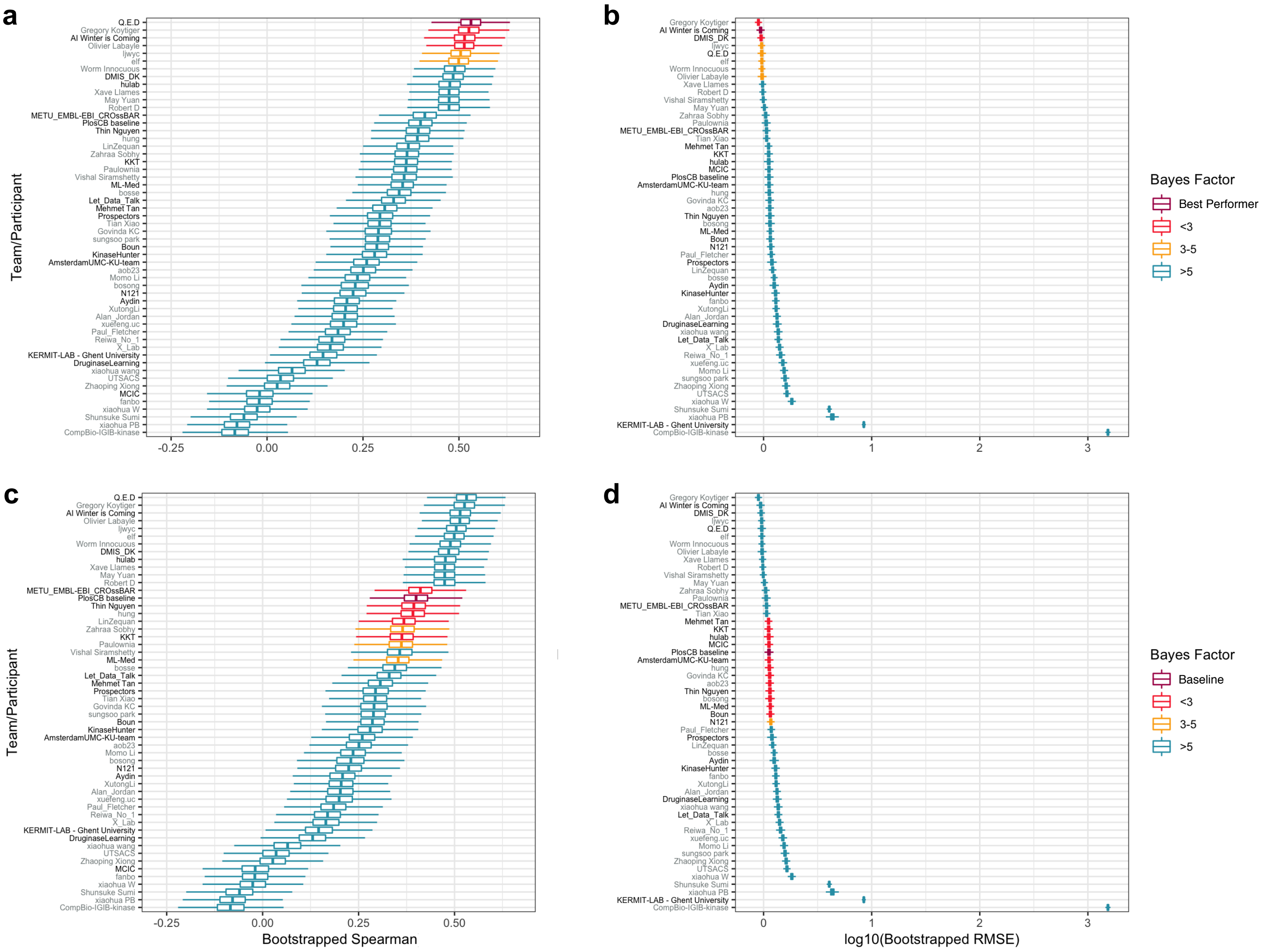

Supplementary Figure 7. Bayes factor analysis of all the participants/teams in Round 2 (qualified participant/team names written in black font). Upper panel: comparison against the top-performing model using (a) bootstrapped Spearman correlation and (b) bootstrapped RMSE. Lower panel: comparison against the baseline model using (c) bootstrapped Spearman correlation and (d) bootstrapped RMSE. Each boxplot summarizes the Spearman correlations or the RMSEs and Bayes factors calculated from n=10000 bootstrapped predictions. In the boxplots, the vertical lines drawn in the middle denote the median, and the left and right hinges correspond to the 25th and 75th percentiles, respectively. The left and right whiskers denote the smallest and largest values, respectively, no further than 1.5 times the inter-quartile range. For visual clarity due to large sample size, the outliers (data points that are not included between the whiskers) are not displayed, but they are included in the Bayes factor calculations. Abbreviation: RMSE, Root Mean Square Error. Source data are provided as a Source Data file (1).

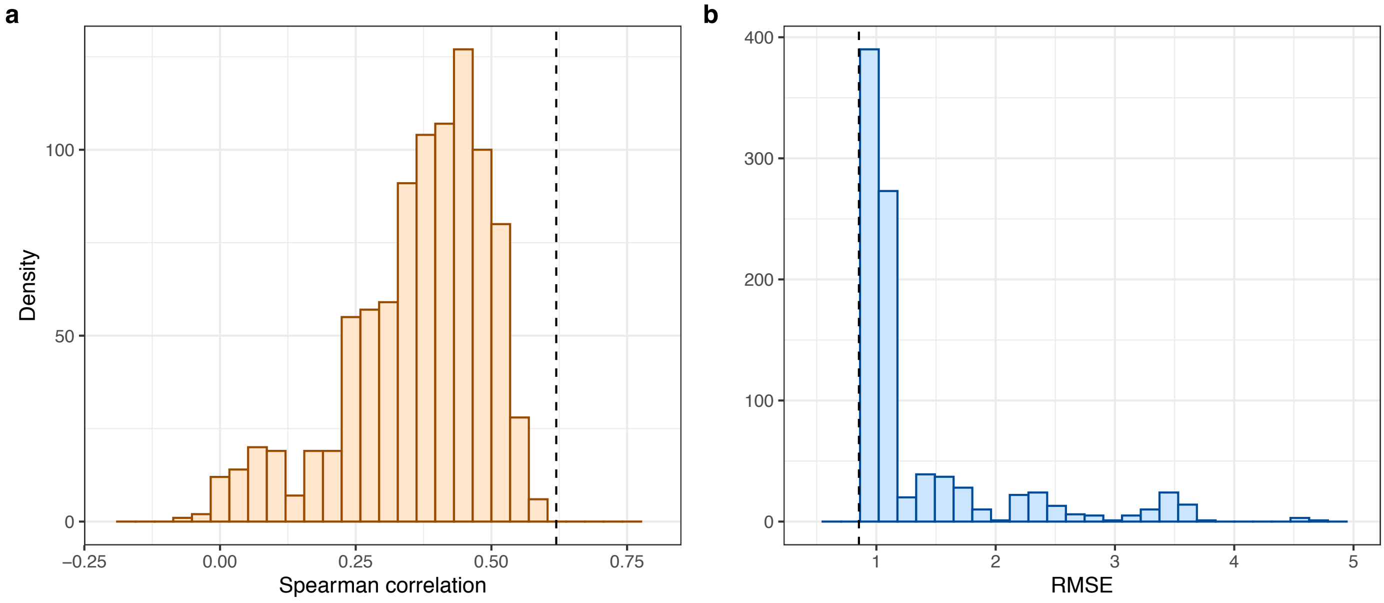

Supplementary Figure 8. Distribution of (a) Spearman correlation coefficient and (b) RMSE of 1000 mean aggregation ensemble models constructed based on randomly sampled sets of four models out of 54 Round 2 submissions. The 73 outlier models (RMSE > 100 and the corresponding negative Spearman correlations) are not displayed. Dashed lines indicate the performance of the mean aggregation ensemble based on the four top-performing models. Abbreviation: RMSE, Root Mean Square Error. Source data are provided as a Source Data file (1).

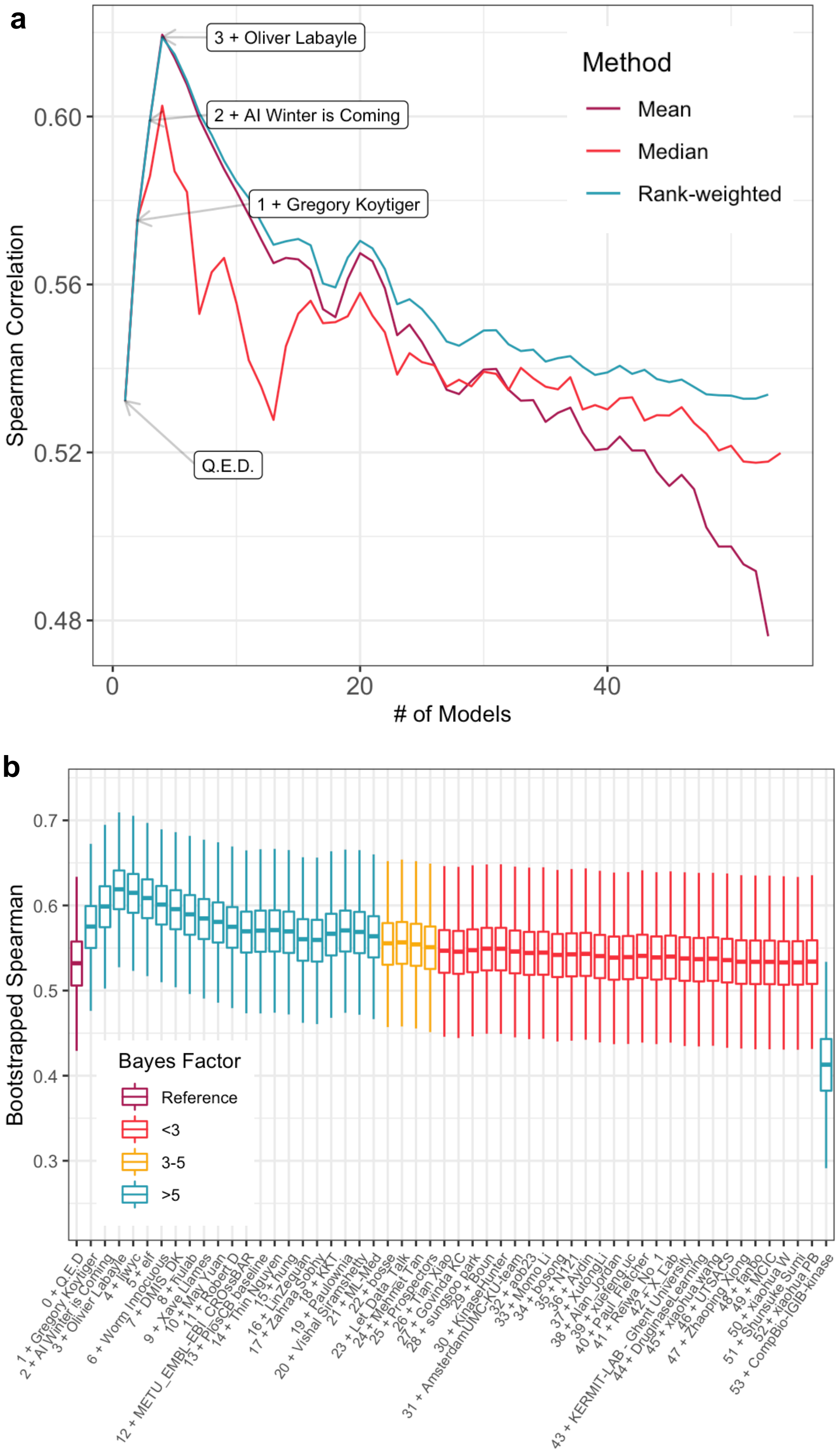

Supplementary Figure 9. Ensemble model construction. (a) Performance of the ensemble models when adding an increasing number of participant models based on their Spearman correlation. Three different model aggregation methods were tested (the color trace legend). (b) Bayes factor analysis of the mean aggregation ensemble model compared to the top-performing reference model (Q.E.D), based on bootstrapped Spearman correlation. Each boxplot summarizes the Spearman correlations and Bayes factors calculated from n=10000 bootstrapped predictions. In the boxplots, the horizontal lines drawn in the middle denote the median, and the lower and upper hinges correspond to the 25th and 75th percentiles, respectively. The lower and higher whiskers denote the smallest and largest values, respectively, no further than 1.5 times the inter-quartile range. For visual clarity due to large sample size, the outliers (data points that are not included between the whiskers) are not displayed, but they are included in the Bayes factor calculations. Source data are provided as a Source Data file (1).

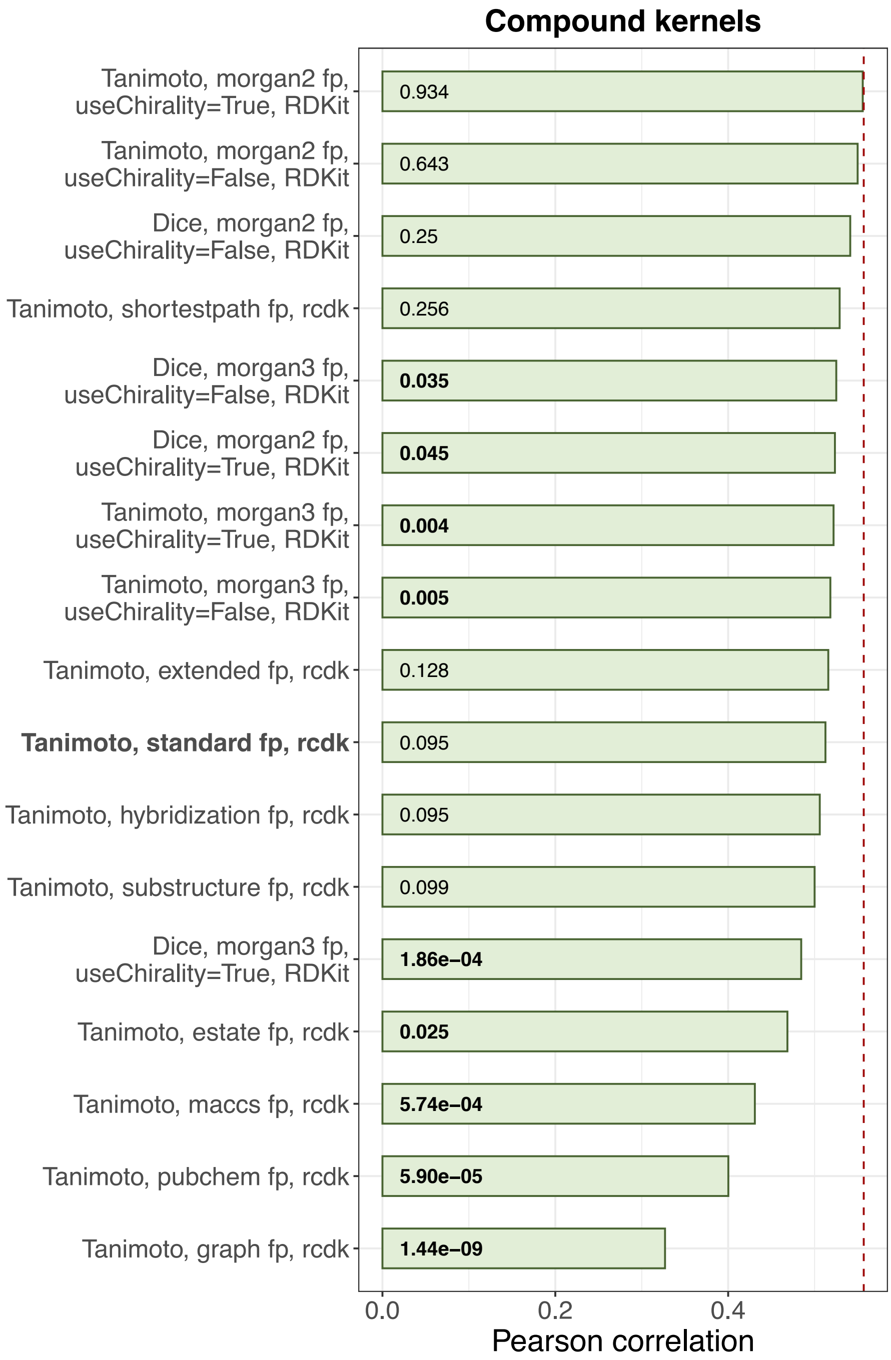

Supplementary Figure 10. Pearson correlations between the measured and Q.E.D model-predicted bioactivities (pK_d's) calculated over 394 Round 2 compound-kinase pairs based on different compound kernels. *P*-values shown inside the bars are Benjamini-Hochberg adjusted and were calculated with the Pearson and Filon test (two-sided) for comparing the correlation of the original, submitted Q.E.D model (with the full amino acid sequence-based Smith-Waterman kernel together with eight RDKit compound kernels) with the correlation of each of the new model variants. Since the two correlations under comparison are calculated on the same set of data points and are overlapping, i.e., they have one variable in common (measured pK_d), also the correlation between pK_d's predicted by the submitted Q.E.D model and the new model variant is taken into account in the statistical test. We note that no other changes were introduced to the submitted Q.E.D model, which is an ensemble of the regressors with different regularisation hyperparameter values and eight compound kernels, but where each regressor is built upon the same protein kernel based on full amino acid sequences. Significant *P*-values are written in boldface. The red dashed line represents the Pearson correlation of the submitted Q.E.D model. The kernel used in the baseline Challenge model is written in boldface. Abbreviation: fp, fingerprint. Source data are provided as a Source Data file (1).

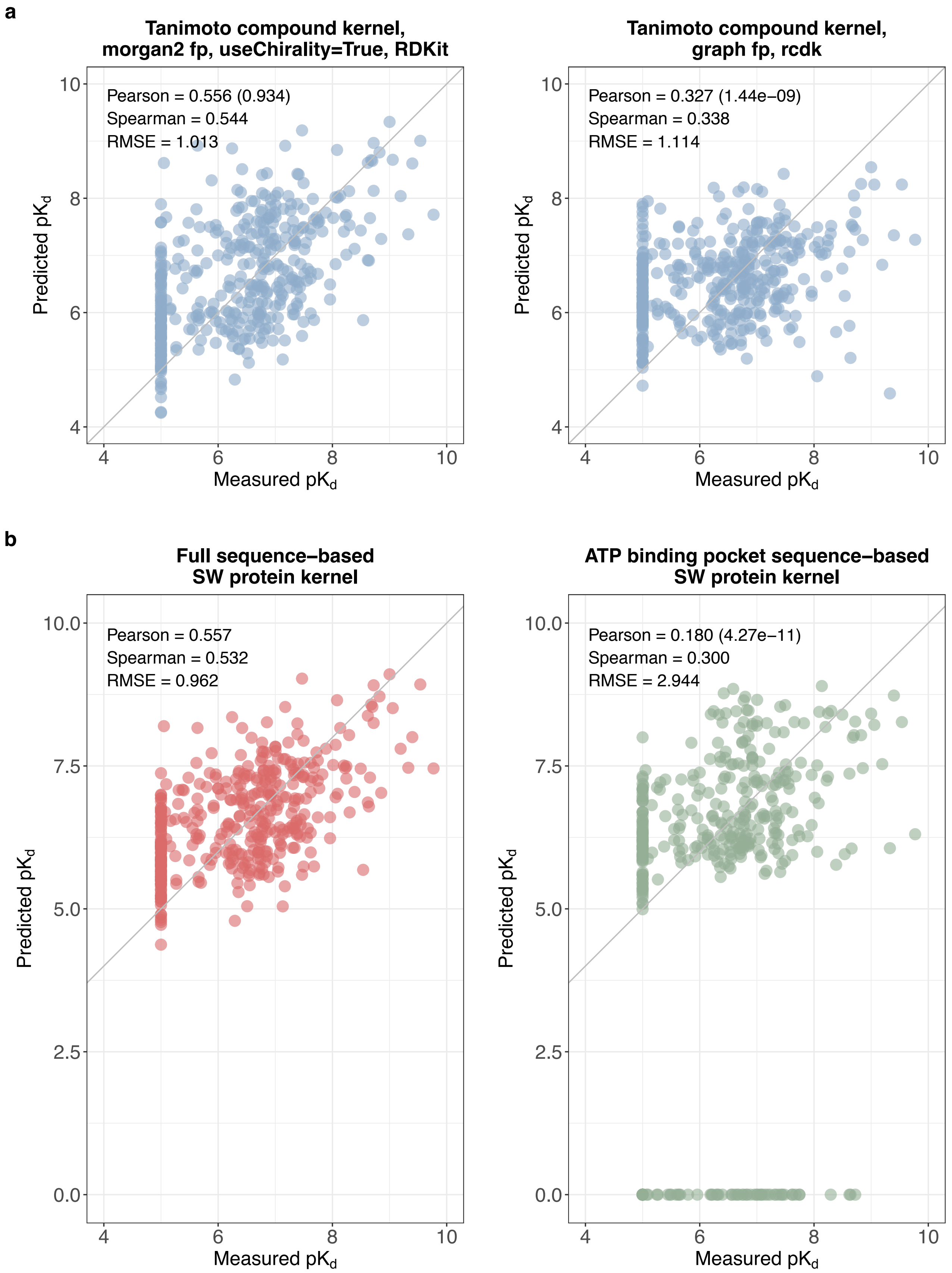

Supplementary Figure 11. Scatter plots between 394 measured and predicted Round 2 pK$_d$ values based on a Q.E.D model run with the best (left panel) and worst (right) performing (a) compound kernels and (b) protein kernels (selected based on Pearson correlation, see Figure 6a and Supplementary Figure 10). The pK$_d$ predictions of zero in panel b correspond to compound-kinase pairs for which kinase amino acid sequences of ATP binding pockets were not available. The model on the left-hand-side of panel b (red) corresponds to the original Q.E.D model submitted to the Challenge. The numbers in parentheses indicate Benjamini-Hochberg adjusted two-sided *P*-values calculated with the Pearson and Filon test for comparing the correlation of the submitted Q.E.D model and each of its re-trained variants. Abbreviations: RMSE, Root Mean Square Error; fp, fingerprint; SW, Smith-Waterman. Source data are provided as a Source Data file (1).

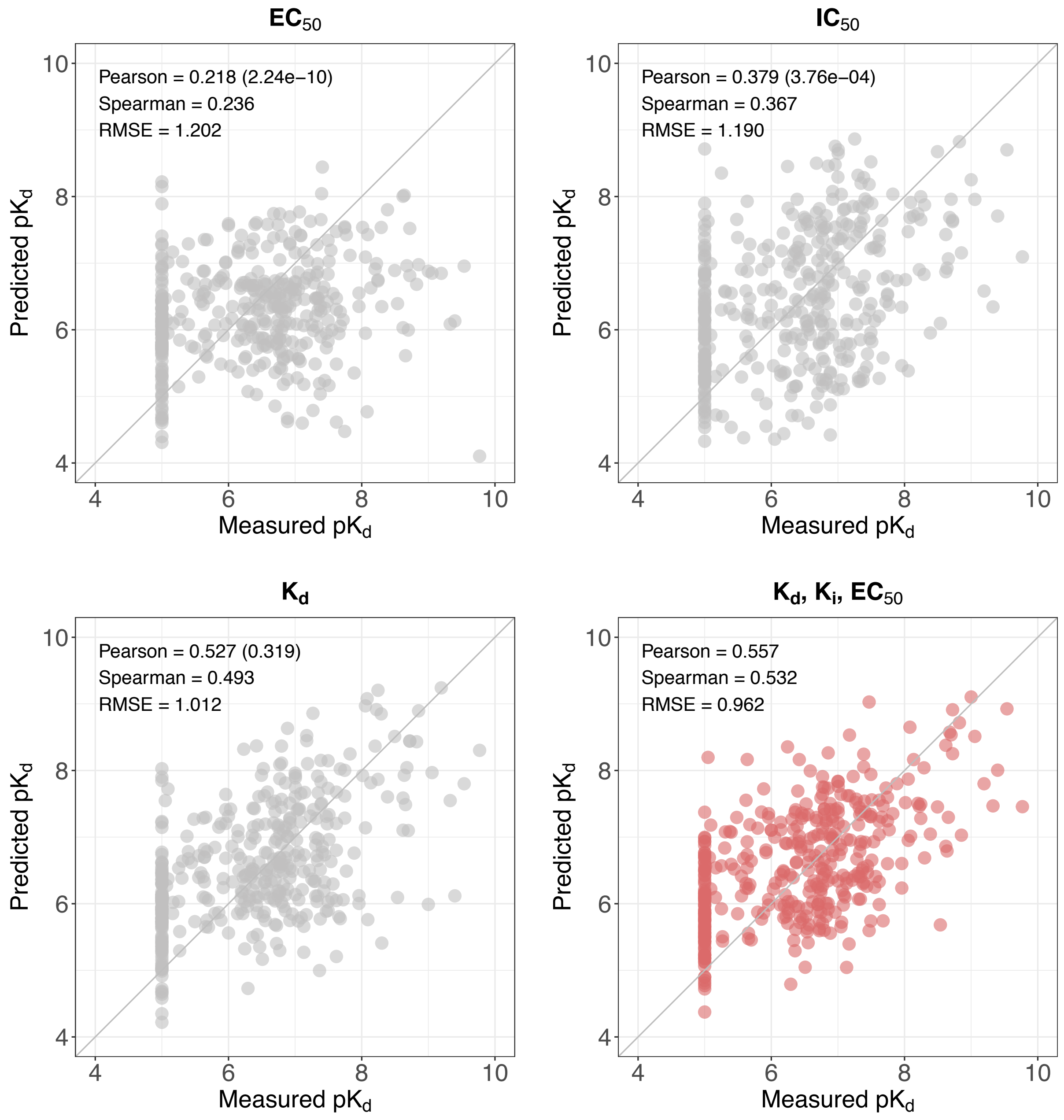

Supplementary Figure 12. Scatter plots between 394 measured and predicted Round 2 pK_d values based on a Q.E.D model with different selected training bioactivity data types (EC_{50}, IC_{50}, K_d, and the original, submitted model that uses a combination of K_d, K_i and EC_{50} bioactivities, marked with red). The numbers in parentheses indicate Benjamini-Hochberg adjusted two-sided *P*-values calculated with the Pearson and Filon test for comparing the correlation of the submitted Q.E.D model and each of its re-trained variants. Abbreviation: RMSE, Root Mean Square Error. Source data are provided as a Source Data file (1).

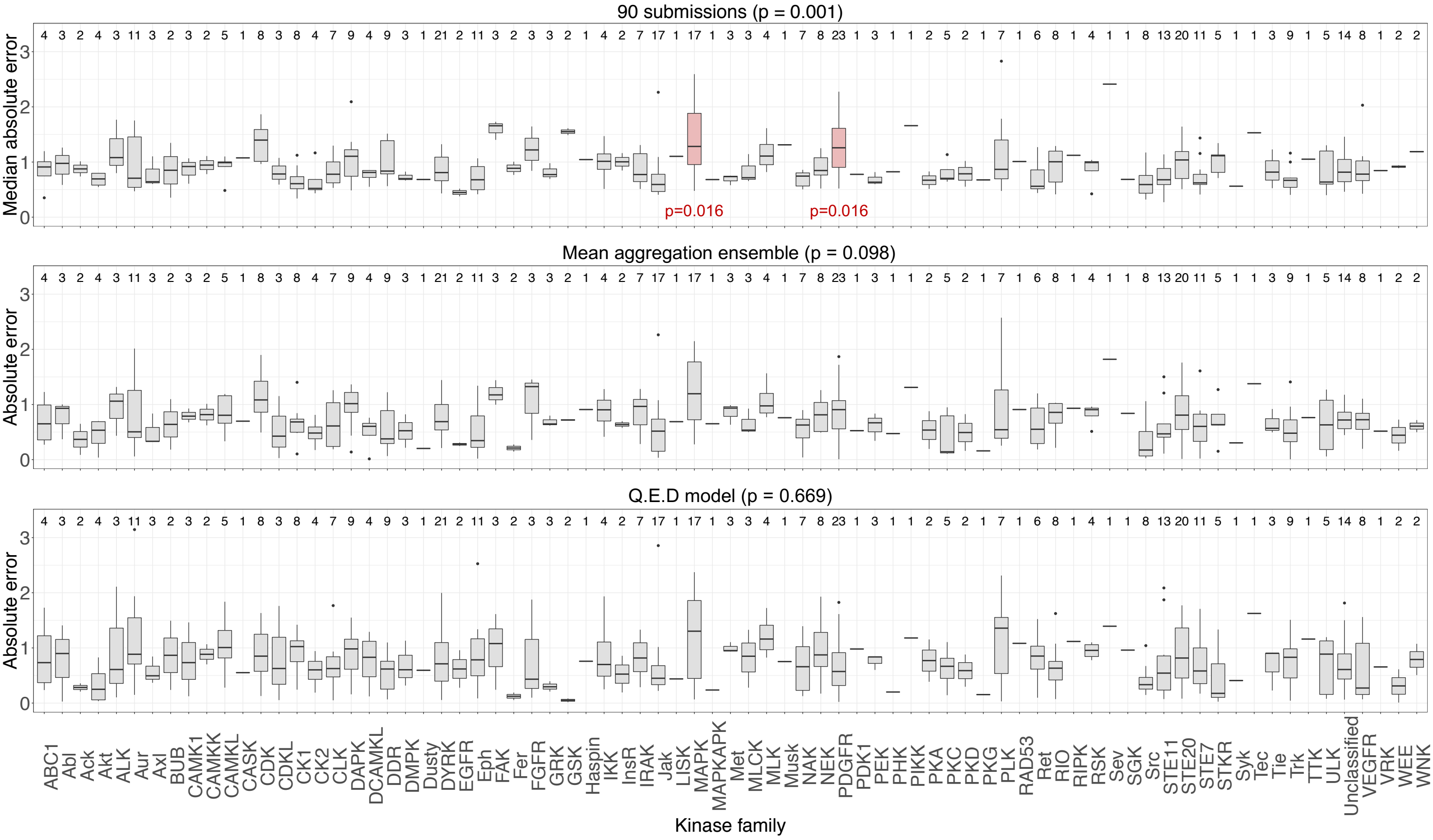

Supplementary Figure 13. Kinase family enrichment analysis results. The overall *P*-values in the title of each plot were calculated based on Kruskal-Wallis test (one-sided), and the group-specific Benjamini-Hochberg-adjusted one-sided *P*-values (only significant *P*-values, in red, are displayed) were calculated based on a weighted Kolmogorov-Smirnov-like statistic, similar to gene set enrichment analysis (see Methods for details). The values at the top of each plot indicate the number of compound-kinase pairs in each kinase family in the Round 2 dataset. The first panel considers 90 out of 99 Round 2 submissions with average absolute error below 2 over the 394 pairs in Round 2, based on which median absolute error was calculated and visualized. In the boxplots, the horizontal lines drawn in the middle denote the median, and the lower and upper hinges correspond to the 25th and 75th percentiles, respectively. The lower and higher whiskers denote the smallest and largest values, respectively, no further than 1.5 times the inter-quartile range. The points that are not included between the whiskers are outliers. Source data are provided as a Source Data file (1).

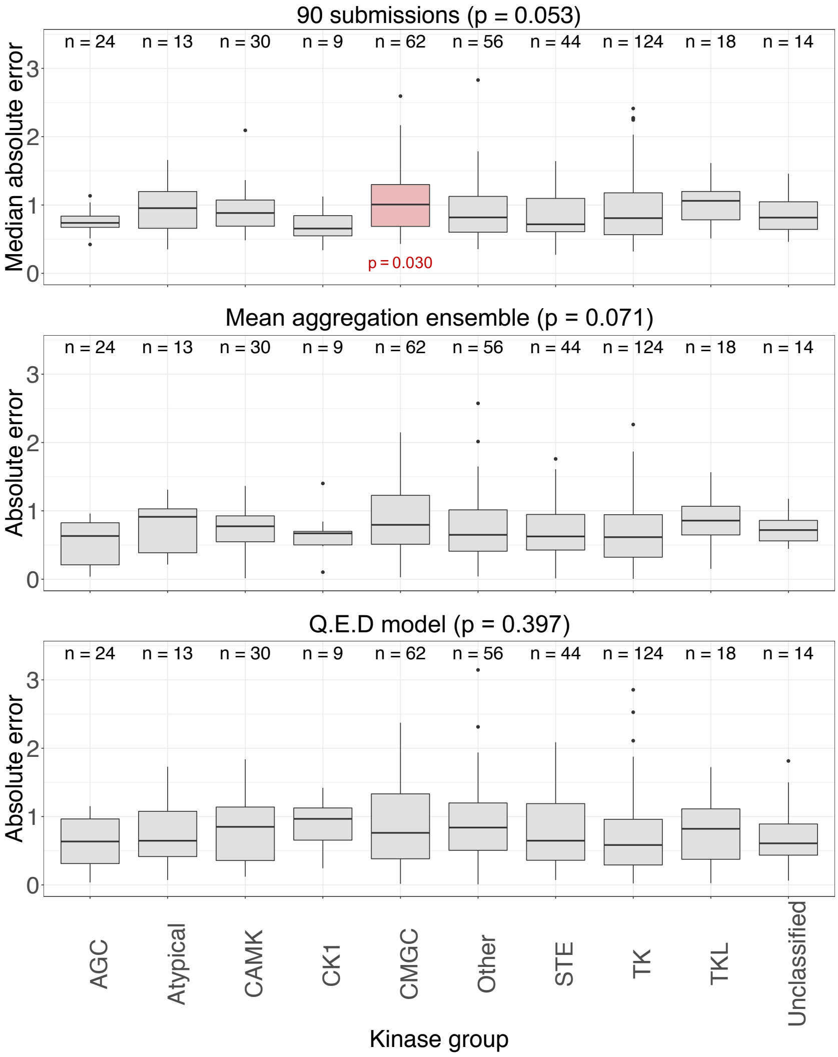

Supplementary Figure 14. Kinase group enrichment analysis results. The *P*-values on top of each panel were calculated based on Kruskal-Wallis test (one-sided), and the group-specific Benjamini-Hochberg-adjusted one-sided *P*-values (only significant *P*-values, in red, are displayed) were calculated based on a weighted Kolmogorov-Smirnov-like statistic, similar to gene set enrichment analysis (see Methods for details). The n values indicate the number of compound-kinase pairs in each kinase group in the Round 2 dataset. In the boxplots, the horizontal lines drawn in the middle denote the median, and the lower and upper hinges correspond to the 25th and 75th percentiles, respectively. The lower and higher whiskers denote the smallest and largest values, respectively, no further than 1.5 times the inter-quartile range. The points that are not included between the whiskers are outliers. Source data are provided as a Source Data file (1).

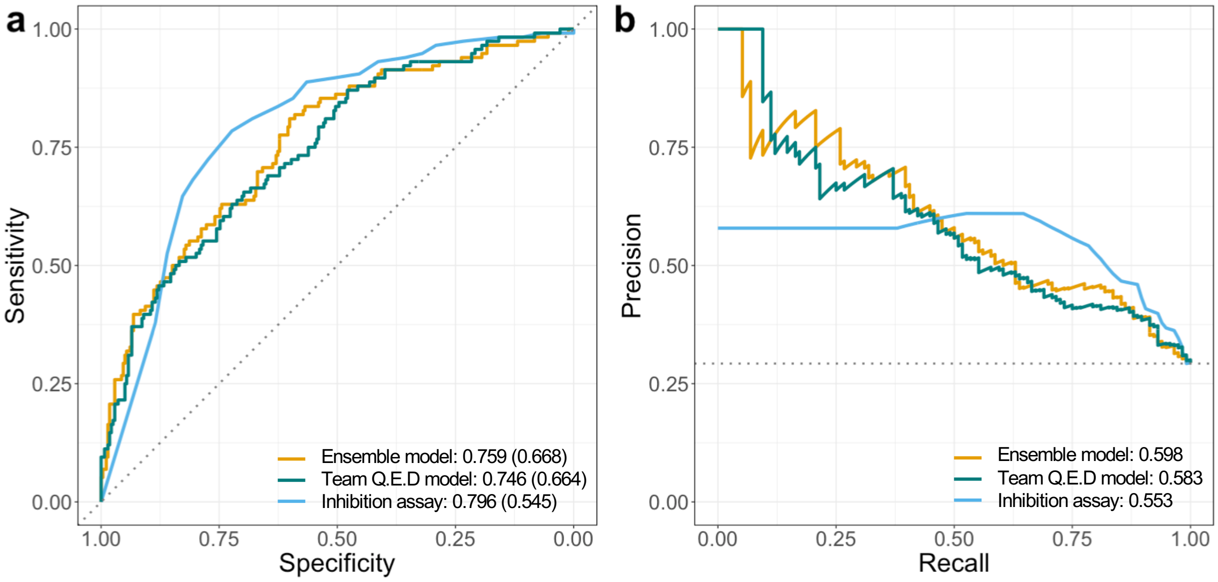

Supplementary Figure 15. (a) Receiver operating characteristic (ROC) curves when ranking the 394 pairs from Round 2 using the ensemble of the top-performing models (average predicted pK_d), top performing Q.E.D model and the single-dose inhibition assays (the true positive activity class includes pairs with measured pK_d>7). The area under ROC curve values are shown in the legend after the predictors, and balanced accuracies are shown in parentheses. The diagonal dotted line shows the random prediction accuracy of AU-ROC=0.50. (b) Precision-recall (PR) curves for the same classification analysis as shown in panel a. The area under the PR curve values are shown in the legend, and the horizontal dotted line indicates the random classifier precision of 0.29. Source data are provided as a Source Data file (1).

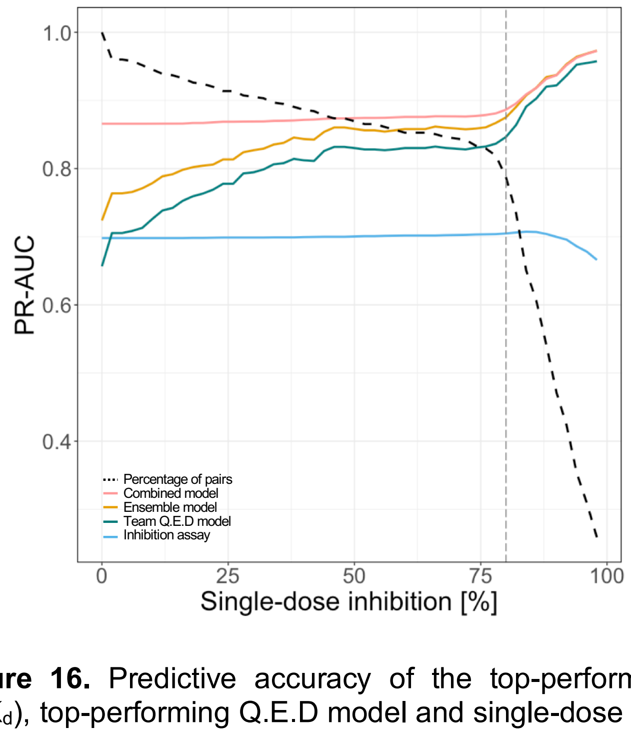

Supplementary Figure 16. Predictive accuracy of the top-performing ensemble model (average predicted pK_d), top-performing Q.E.D model and single-dose assay (at 1 μM), when classifying subsets of the 475 pairs into the true activity classes with measured pK_d less or higher than 6. The y-axis indicates the area under the precision-recall (PR) curve (PR-AUC) as a function of the single-dose inhibition% levels, x-axis the pairs with inhibition>x%, and the dotted black curve the percentage of all pairs that passed that single-dose activity threshold. The combined model trace corresponds to the average of measured and expected inhibition values, where the latter was calculated based on the mean ensemble of the top-performing model pK_d predictions (Q.E.D, DMIS_DK and AI Winter is Coming). Source data are provided as a Source Data file (1).

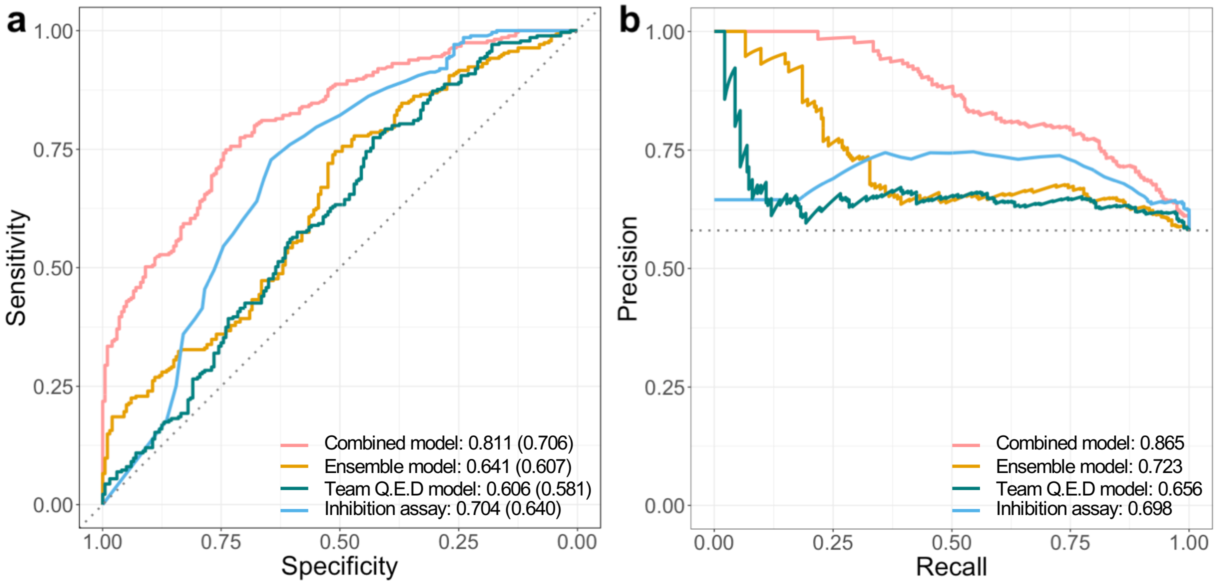

Supplementary Figure 17. Receiver operating characteristic (ROC) curves and (b) precision-recall (PR) curves when ranking all the 475 compound-kinase pairs using either the top-performing ensemble model-predicted pK_d values, the measured single-dose inhibition assays, or their combination. The performance of using the top-performing Q.E.D model-predicted pK_d values is also shown. The true positive activity class contains pairs with measured $pK_d > 6$. (a) The area under the ROC curve values are shown after the predictors (and the balanced accuracy in the parentheses), and the diagonal dotted line shows the random predictor with an accuracy of AU-ROC=0.50. (b) The area under the PR curve values are shown after the predictors and the horizontal dotted line indicates the random predictor with a precision value of 0.58. Source data are provided as a Source Data file (1).

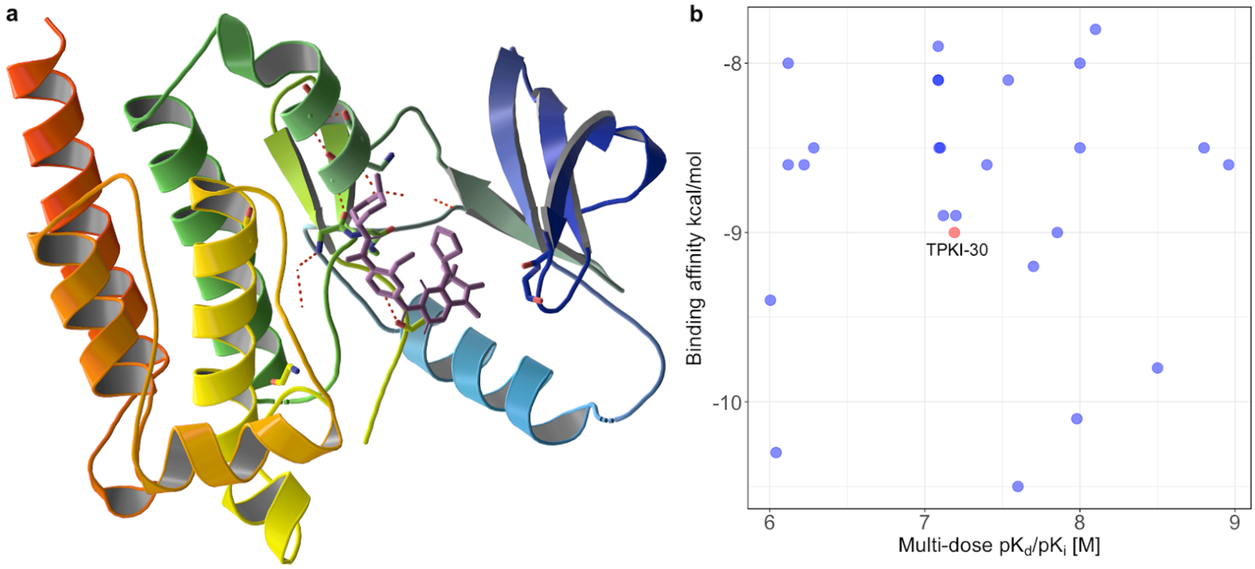

Supplementary Figure 18. (a) A receptor-ligand complex from the docking that shows the optimal pose resulting from the binding of a compound TPKI-30 to PYK2 protein structure (5TO8). The likely interaction between the ligand and side chains of the PYK2 target is marked in red. (b) The binding affinity predictions of various active ligands used in the docking study in terms of their measured pK_d/pK_i activity values. Even though there is no significant correlation between the binding affinity and the compound activity, TPKI-30 showed a binding energy value of -9.1 kcal/mol (marked in red), similar to many other known active ligands of PYK2 (blue points). The docking was done with AutoDock Vina. The X-ray crystal structure of protein PYK2 (5TO8) was obtained from RSCB, and a collection of 26 compounds (including TPKI-30), with potent activity towards PYK2 (i.e., $pK_d/pK_i > 6$) from DTC, ChEMBL and Binding DB, were used as ligands in the docking procedure. Source data are provided as a Source Data file (1).

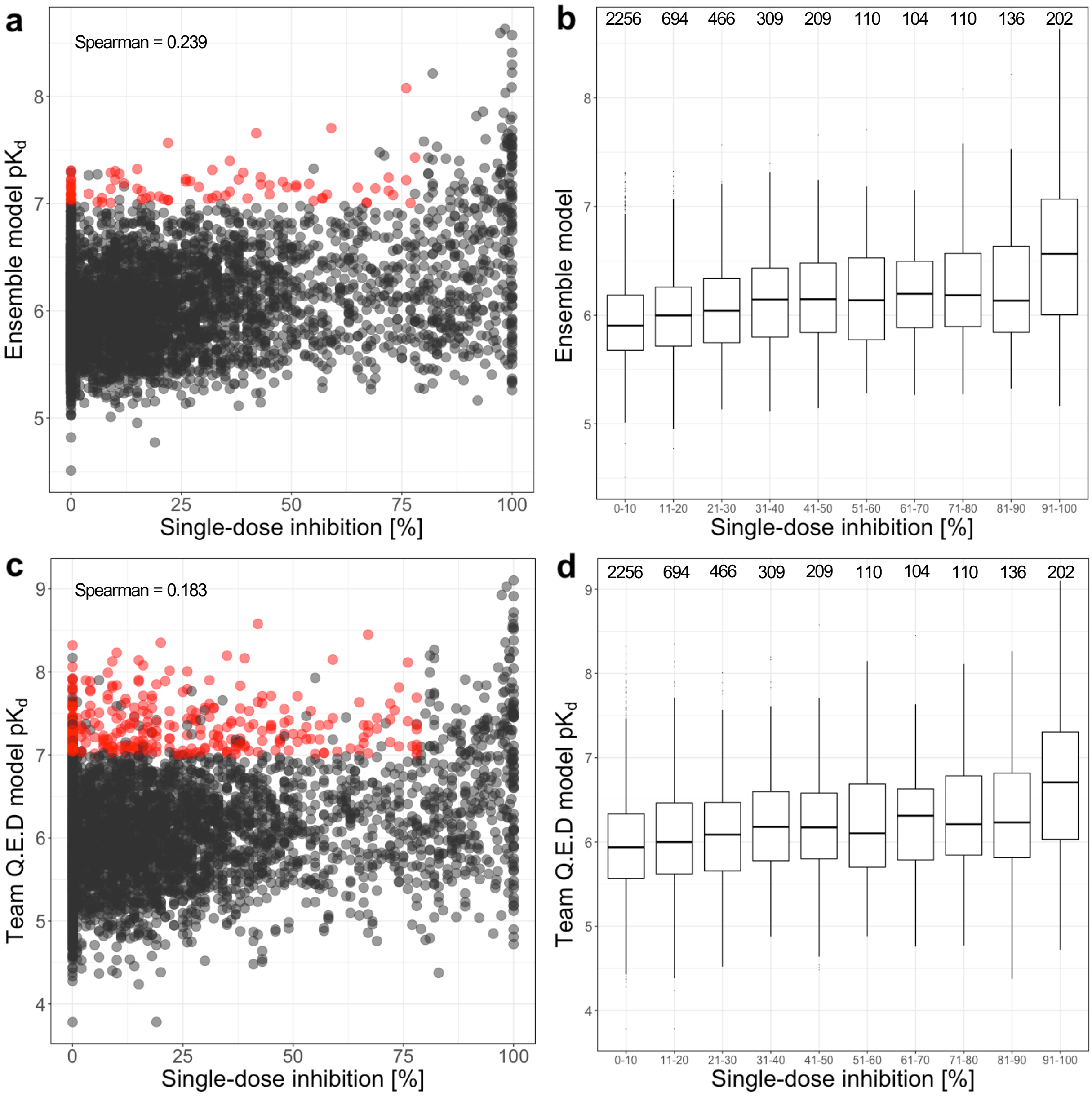

Supplementary Figure 19. (a, c) Average pKd prediction of (a) the top-performing models' ensemble and (c) top-performing Q.E.D model against single-dose inhibition levels in the full Round 2 data matrix, consisting of 5100 pairs between 25 inhibitors and 204 kinases that had %inhibition measurements available. Interestingly, the prediction models did not use any single-dose activity data in their training, and therefore showed only a marginal correlation with the measured %inhibition levels. (b, d) The same data analyzed using box plots to better show the relationships at the lowest (0%) and highest (100%) single-dose inhibition levels. The red points indicate potential false negatives based on the single-dose inhibition assay that have relatively high predicted $pK_d>7$ but were not K_d profiled for the Round 2 dataset due to relatively low single-dose inhibition assay activity. The values on top of each box-whiskers indicate the number of compound-kinase pairs with %inhibition values in specified inhibition range given in the x-axis. In the boxplots, the horizontal lines drawn in the middle denote the median, and the lower and upper hinges correspond to the 25th and 75th percentiles, respectively. The lower and higher whiskers denote the smallest and largest values, respectively, no further than 1.5 times the inter-quartile range. The points that are not included between the whiskers are outliers. Source data are provided as a Source Data file (1).

a

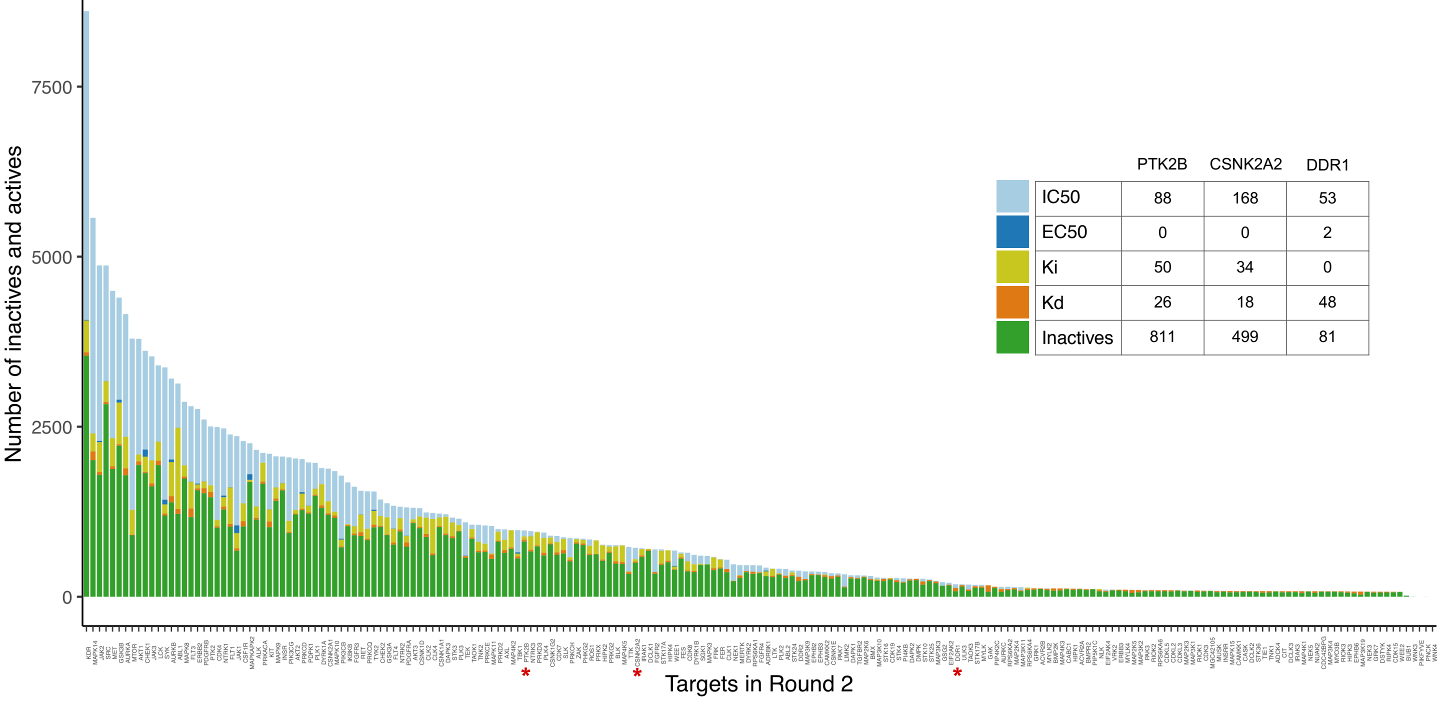

b

Multi-dose bioactivity type	Number of Q.E.D training data points for PTK2B	Average number of Q.E.D training data points for DDR1	Average number of Q.E.D training data points for CSNK2A2	Average number of Q.E.D training data points for Round 2 kinases	Maximum number of Q.E.D training data points for Round 2 kinases
All	89	56	52	167	1047
K_d	26	52	25	31	136
K_i	63	2	27	131	976
EC_{50}	0	2	0	5	147

Supplementary Figure 20. (a) The number of bioactivity data points available across 207 kinase targets in the Round 2 dataset of the Challenge. PYK2 (PTK2B), CSNK2A2 and DDR1 are marked with an asterisk, and the numbers of their bioactivity data points are shown in a table in the inset. The dose-response bioactivity data were extracted from DTC, ChEMBL, canSAR, DrugKiNET and IUPHAR databases. The active and inactive compound-kinase interactions were classified based on the fixed activity threshold of 1000nM. (b) The number of training bioactivity values behind the Q.E.D model for the prediction of the interaction between PFE-PKIS 10 and PYK2 as well as multiple interactions with DDR1 and CSNK2A2 kinases in the Round 2. The average and maximum number of training data points across all the 207 test kinases in Round 2 are also shown as comparison reference values. Source data are provided as a Source Data file (1).

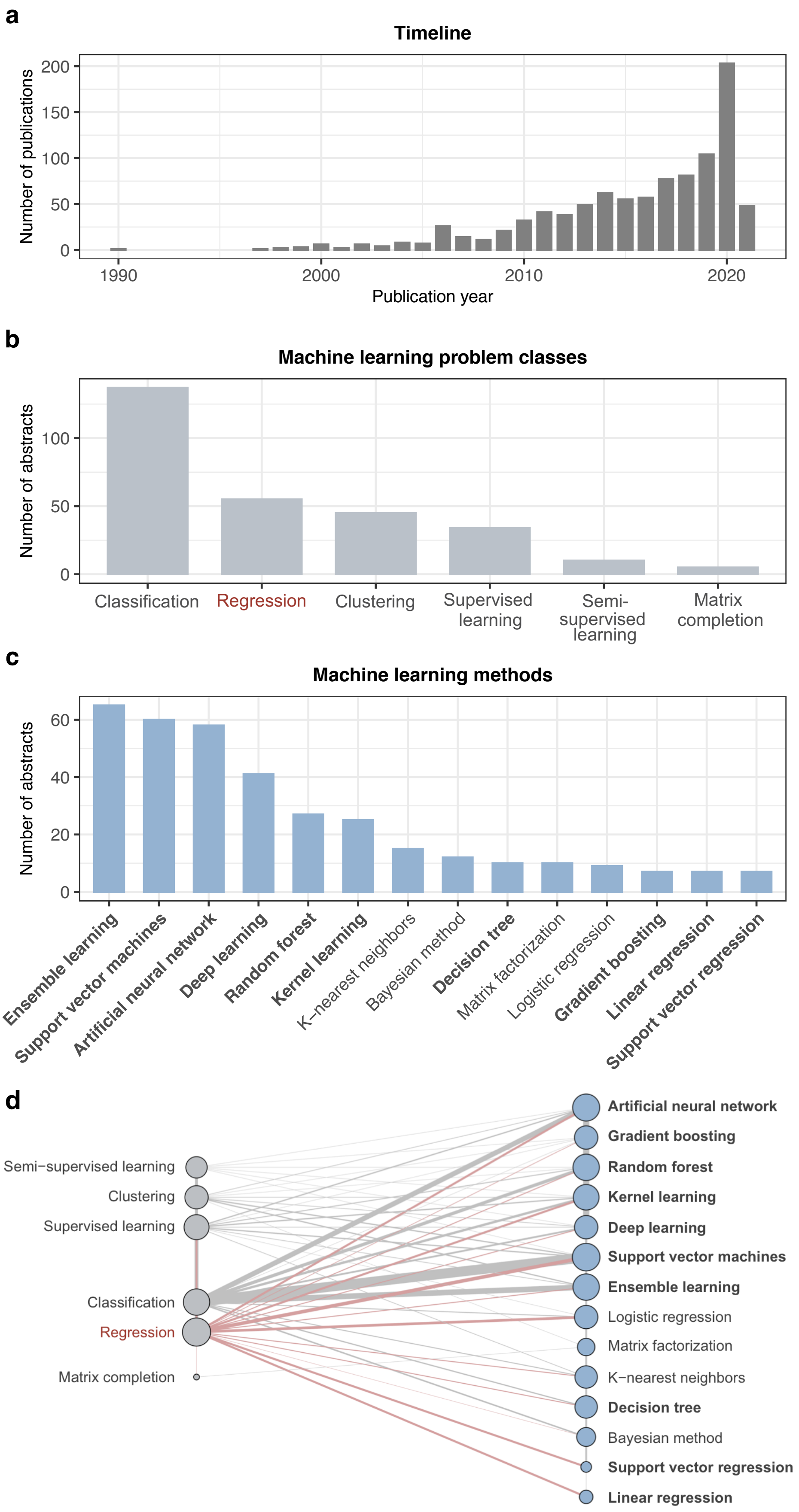

Supplementary Figure 21. (a) Number of PubMed publications on compound-target interaction prediction as of 16 February 2021; see Methods for keywords. (b, c) Number of publication abstracts mentioning different (b) machine learning problem classes and (c) machine learning methods. (d) Co-occurrence graph of problem classes and machine learning methods in 959 PubMed abstracts. The edge weight indicates the frequency of the co-occurrence in the literature, and the node size is proportional to the degree of networking, i.e., the number of connections the node has to other machine learning approaches and problem classes. The methods used by the Challenge teams are boldfaced and the Challenge problem task is indicated in red text. Source data are provided as a Source Data file (1).

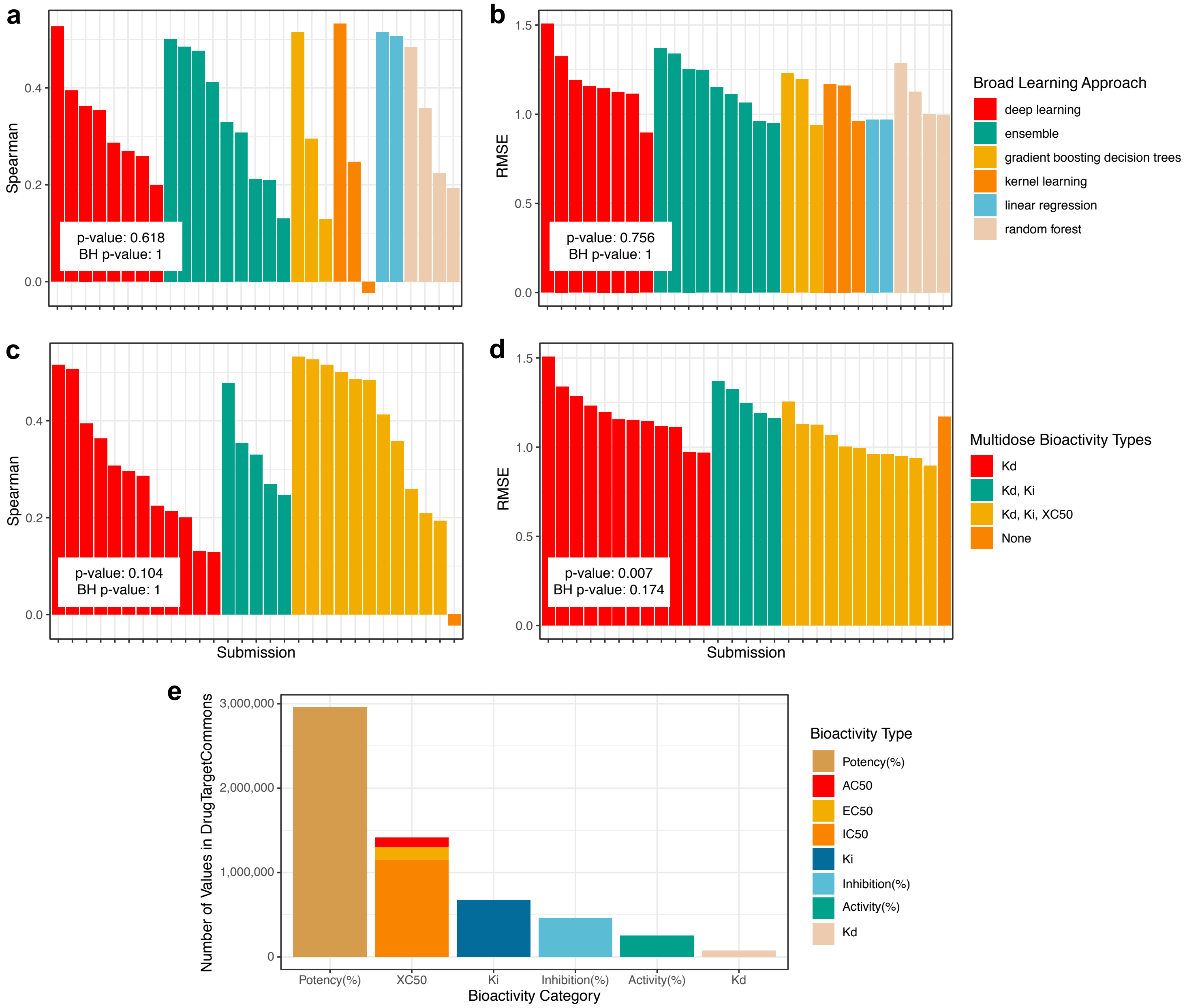

Supplementary Figure 22. The survey questionnaire results concerning the (a, b) broad learning approaches and (c, d) multi-dose bioactivity data types used in the model training. Statistical significance was assessed using the Kruskal-Wallis test (unadjusted one-sided *P*-values), and adjusted with Benjamini-Hochberg (BH) control of false discovery rate (FDR). (e) Number of bioactivity values available in DrugTargetCommons for different bioactivity types. Source data are provided as a Source Data file (1).

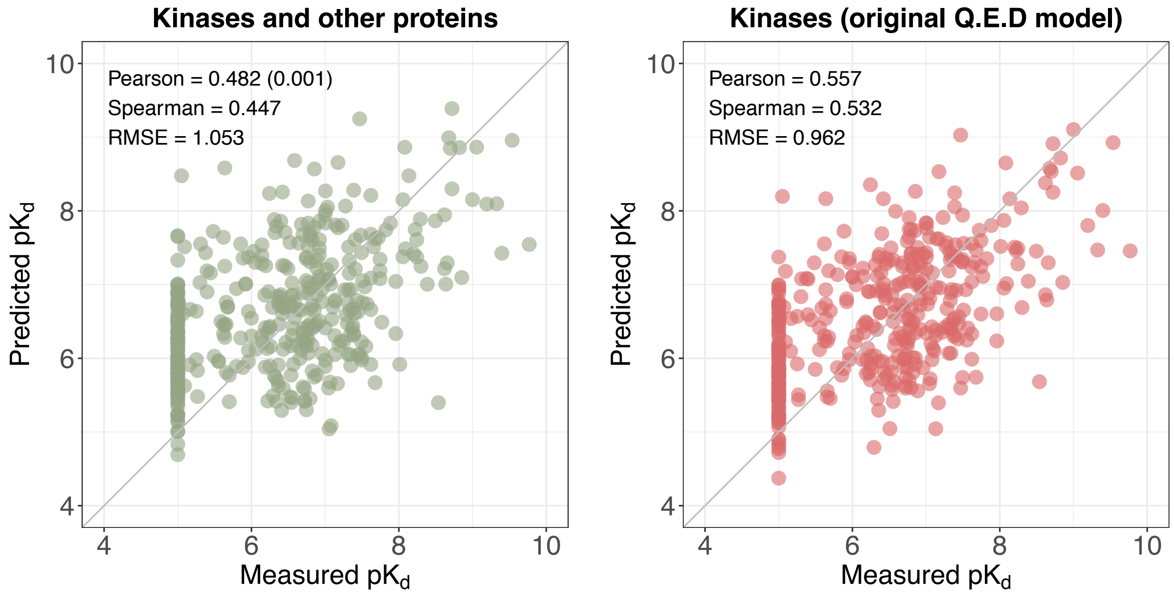

Supplementary Figure 23. Scatter plots between 394 measured and predicted Round 2 pK_d values based on the original Q.E.D model that included only kinases in the training data (red) and the new Q.E.D model that includes bioactivities (K_d, K_i and EC_{50}) for kinases and other proteins. Specifically, Q.E.D model additionally incorporated training bioactivity data extracted from Drug Target Commons for G-protein-coupled receptors (GPCRs), ion channels, nuclear receptors, transcription factors and transporter proteins (ca. 100 000 compounds, out of which ca. 10 000 were kinase inhibitors). This increased the number of protein targets in the training dataset from roughly 530 to 900, including both kinases and other proteins, but the number of compounds in the prediction model was kept similar as in the original submitted model by selecting ca. 13 600 of 100 000 compounds most similar (based on Tanimoto similarity of Morgan fingerprints) to the ones in the Challenge test data (4 197 kinase inhibitors and 9 436 other compounds). The number in parentheses indicates two-sided *P*-value calculated with the Pearson and Filon test for comparing the correlation of the original Q.E.D model and the newly-tested model. Abbreviation: RMSE, Root Mean Square Error. Source data are provided as a Source Data file (1).

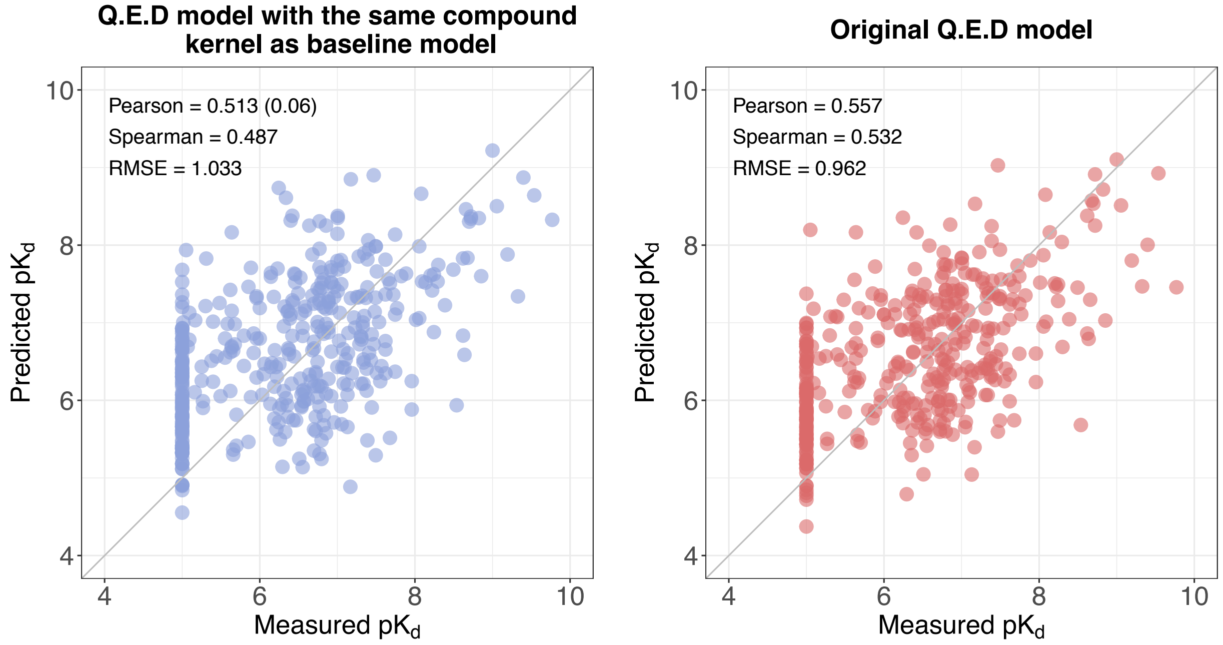

Supplementary Figure 24. Scatter plots between 394 measured and predicted Round 2 pK_d values based on the original Q.E.D model (red), and a modified model including the same compound kernel as the baseline model (kernel calculated based on path-based 1024-bit fingerprints from the rcdk R package; otherwise the Q.E.D model remained the same). The number in parentheses indicates two-sided *P*-value calculated with the Pearson and Filon test for comparing the correlation of the original Q.E.D model and the newly-tested model. Abbreviation: RMSE, Root Mean Square Error. Source data are provided as a Source Data file (1).

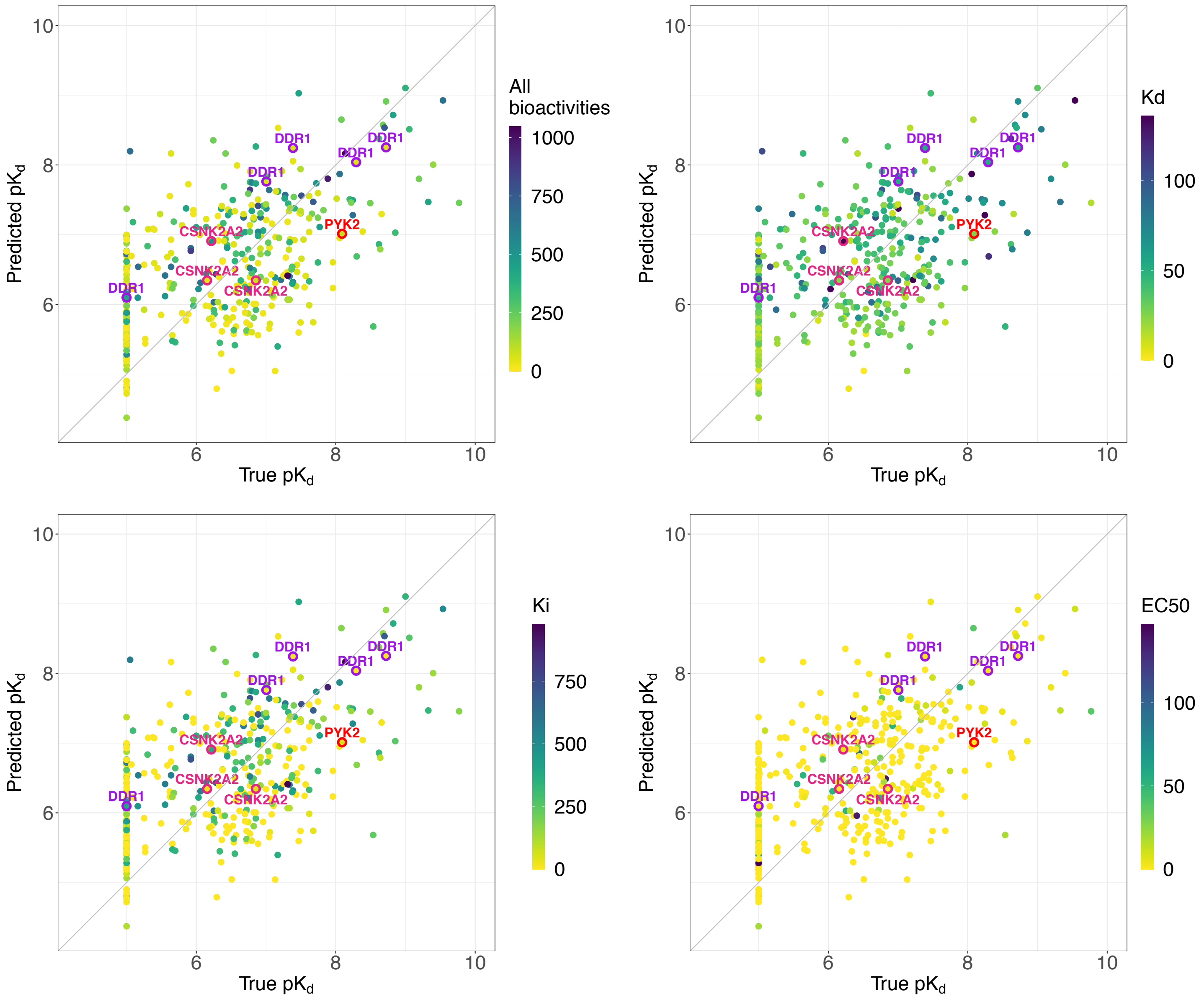

Supplementary Figure 25. Prediction accuracy of the top-performing model (Q.E.D) in Round 2. The points correspond to the 394 pairs between 25 compounds and 207 kinases, and the color in each panel indicates the number of training data points for each pair from different bioactivity types. The compound-kinase pairs involving PYK2, DDR1 and CSNK2A2 kinases are highlighted. Source data are provided as a Source Data file (1).

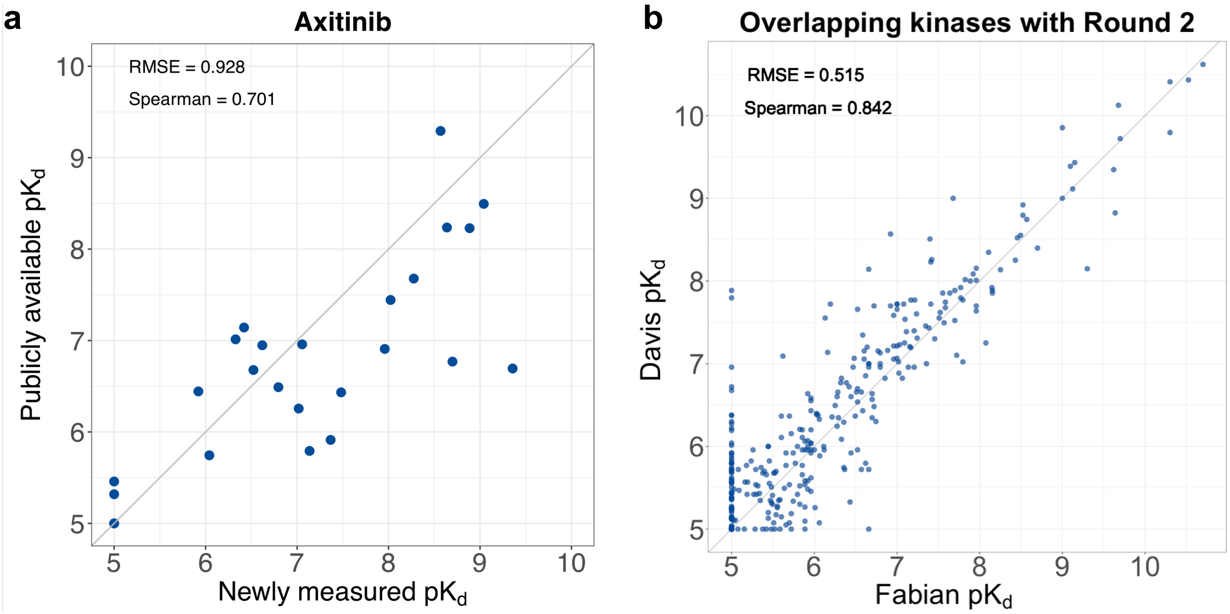

Supplementary Figure 26. (a) pK_d values of the 25 axitinib-kinase pairs generated for Round 2 compared to those available in DTC. (b) pK_d values of 412 compound-kinase pairs for kinases that overlapped with the Round 2 kinases from two comprehensive target profiling studies carried out by Davis et al. (2) and Fabian et al. (3). Out of the 412 pairs, 103 pairs have pK_d=5 in both of the studies, leading to relatively low RMSE. Abbreviation: RMSE, Root Mean Square Error. Source data are provided as a Source Data file (1).

Supplementary Table 1. Model classes and training data of the baseline model and models submitted to Round 2 together with method surveys sorted by Spearman correlation. Even if teams chose to combine predictions from multiple models, they had to submit only one prediction per each compound-target pair for scoring against the measured activities. The entries corresponding to Challenge baseline model are written in boldface type.

Team	Algorithm type	Algorithm name	Combined models	Training strategy	Training data sources	Compound-protein pairs	Bioactivity types	Protein features	Chemical features	Spearman correlation	RMSE
Q.E.D	Kernel learning	CGKronRLS	440	Boosting	DrugTargetCommons, ChEMBL, UniProt	60462	K_d, K_i, EC_{50}	Amino acid sequences	ECFP	0.532	0.962
Gregory Koytiger	Deep learning, artificial neural network	-	6	Fixed hold out	ChEMBL	250000	K_d, K_i, IC_{50}	Amino acid sequences	SMILES strings	0.527	0.897
AI Winter is Coming	Gradient boosting decision trees	XGboost	5 per target	K-fold nested cross validation, boosting	DrugTargetCommons, ChEMBL	600000	K_d, K_i, IC_{50}, EC_{50}, %inh, %activity	None	ECFP	0.515	0.939
Olivier Labayle	Ridge regression	-	-	K-fold cross validation	DrugTargetCommons, ChEMBL, UniProt	18200	K_d	K-mer counting	ECFP	0.515	0.970
Olivier Labayle	Ridge regression	-	-	K-fold cross validation	DrugTargetCommons, ChEMBL, UniProt	18000	K_d	K-mer counting	ECFP	0.507	0.970
DMIS_DK	Deep learning, multi-target learning	Multi-Task Graph Convolutional Neural Networks	12	Train test split	DrugTargetCommons, BindingDB	953521	K_d, K_i, IC_{50}	None	Molecular graphs	0.485	0.949
DMIS_DK	Random forest	-	-	Boosting	DrugTargetCommons, BindingDB	128181	K_d, K_i, IC_{50}	None	Morgan fingerprint	0.484	1.002
hulab	Stacked model, gradient boosting	Stacked model using XGB, catboost, LGBM and Ridge regressors	22	Boosting	DrugTargetCommons, ChEMBL, Drewry et al. (4)	52588	K_d, K_i,%inh, %activity	Kinase domain sequences and kinase to kinase correlation based on substrates	Morgan, pharmacophore distance features, SMILES-based autoencoder features	0.477	1.248
METU_EMBL-EBI_CROssBAR	Deep learning, artificial neural network, multi-target learning	Feed-forward deep neural networks	-	K-fold cross validation	ChEMBL	192935	K_d, K_i, IC_{50}	2D (tri-gram-PSSM and k-separatedbigram-PSSM)	ECFP	0.412	1.066
**Baseline**	**Kernel learning**	**CGKronRLS**	**1**	**K-fold nested cross validation**	**DrugTargetCommons**	**44186**	**K_d**	**Amino acid sequences**	**Path-based fingerprints**	**0.400**	**1.123**
thinng	Deep learning	-	-	K-fold cross validation	DrugTargetCommons	75185	K_d	None	SMILES strings	0.394	1.146
KKT	Deep learning	DeepAffinity	30	K-fold cross validation	BindingDB, STITCH, UniRef	17819	K_d	Amino acid sequences, physicochemical properties	SMILES strings	0.363	1.116
siramshettyv2	Random forest	-	-	K-fold cross validation	ChEMBL	93908	K_d, K_i, IC_{50}, EC_{50}, AC_{50}	None	ECFP	0.358	0.994
ML-Med	Deep learning, transfer learning	-	-	K-fold cross validation	DrugTargetCommons, ChEMBL	2185412	K_d	Amino acid sequences	MACCS	0.353	1.190
Let_Data_Talk	Deep learning, kernel learning	SVM, Attention RNN	6	K-fold cross validation	DrugTargetCommons, ChEMBL	101469	K_d, K_i, %activity	Amino acid sequences	Topological torsion	0.330	1.372
oselot	Deep learning, gradient boosted decision trees	LightBoost	2	Train, validation, test split	DrugTargetCommons	55678	K_d	Amino acid sequences	ECFP	0.307	1.113
Prospectors	Gradient boosting decision trees	XGboost	-	Boosting	DrugTargetCommons	7336	K_d	Amino acid sequences	MACCS	0.300	1.196
Boun	Deep learning	DeepDTA	-	Default hyperparameters used	DrugTargetCommons	50181	K_d	Amino acid sequences	SMILES strings	0.286	1.156
KinaseHunter	Deep learning, artificial neural network	Graph Convolution, Neural Fingerprint, Attention Mechanism	-	Train, validation, test split	ChEMBL, LINCS-HMS KinomeScan database	442489	K_d, K_i	Amino acid sequences	Neural fingerprint	0.270	1.325
AmsterdamUMC-KU-team	Convolutional neural networks	-	-	K-fold cross validation	DrugTargetCommons, ChEMBL, IUPHAR/BPS, literature	298595	K_d, K_i, IC_{50}	Amino acid sequences	ECFP	0.259	1.125
Q.E.D	Kernel learning	CGKronRLS	5184	Boosting	DrugTargetCommons, ChEMBL	58023	K_d, K_i	Amino acid sequences, protein-protein interactions, protein structures, protein-pathway association network, GO annotations	ECFP	0.247	1.161
N121	Random forest	-	-	Train test split	DrugTargetCommons, IDG Pharos	145	K_d,%inh	None	Morgan fingerprint	0.224	1.287
ML-Med	Deep learning, transfer learning	-	-	K-fold cross validation	DrugTargetCommons, ChEMBL	2185412	K_d	Amino acid sequences	MACCS	0.213	1.154
Aydin	Deep learning, multi-target learning	LSTM, Convolutional Neural Networks, Multi-task learning	-	K-fold cross validation	DrugTargetCommons, Davis et al. (2), KIBA	694801	K_d, K_i, IC_{50}	Amino acid sequences	SMILES strings	0.209	1.255
xuefeng	Deep learning	-	3	K-fold cross validation	DrugTargetCommons	14875	K_d	None	None	0.200	1.508
METU_EMBL-EBI_CROssBAR	Random forest	-	-	K-fold cross validation	ChEMBL	94184	K_d, K_i, IC_{50}	2D (tri-gram-PSSM and k-separatedbigram-PSSM)	ECFP	0.193	1.127
KERMIT-LAB	Multi-kernel learning	Two-step kernel ridge regression	-	K-fold cross validation	DrugTargetCommons	57000	K_d	Amino acid sequences	PubChem fingerprint	0.147	8.466
Druginase Learning	Deep learning, transfer learning	LSTM, CNN, Autoencoder	-	K-fold cross validation	DrugTargetCommons, ChEMBL, KIBA, Davis et al. (2), PKIS, KKB, BindingDB, HMS LINCS	68903	K_d	None	SMILES strings	0.131	1.340
Prospectors	Gradient boosting decision trees	XGboost	-	Boosting	DrugTargetCommons	13786	K_d	Amino acid sequences	MACCS	0.129	1.232
MCIV	Kernel learning	-	-	K-fold cross validation	DrugTargetCommons	28403	K_d	Amino acid sequences	PubChem fingerprint	-0.023	1.170
CompBio-IGIB-kinase	Random forest	-	10	K-fold cross validation	DrugTargetCommons	12498	K_d	Amino acid sequences	PubChem fingerprint	-0.083	1534.124

Supplementary Table 2. SEA-predicted Round 2 targets of PFE-PKIS 14, TPKI-30 and GSK1379763. Due to a large number of predictions for TPKI-30 and GSK1379763, only the targets with *P*<1e-10 are listed. The *P*-values are estimated using an empirical statistical model that best-fits the calculated Z-scores to a right-tail Extreme Value Gumbel Distribution. It is a one-sided statistical test and compound-target pairs are independent samples, and hence no *P*-value adjustment was applied.

Compound	SEA-predicted target		
	**Gene symbol**	**UniProt ID**	***P*-value**
PFE-PKIS 14	CHEK2	O96017	1.11e-16
	CHEK1	O14757	2.434e-08
	IKBKB	O14920	4.867e-08
	FLT1	P17948	3.819e-07
TPKI-30	PTK2	Q05397	4.626e-121
	CDK4	P11802	3.747e-38
	PLK1	P53350	4.647e-37
	NEK3	P51956	4.752e-33
	ALK	Q9UM73	4.345e-29
	BMX	P51813	1.337e-24
	CSF1R	P07333	5.055e-21
	TTK	P33981	9.975e-21
	NIM1K	Q8IY84	1.463e-20
	PLK2	Q9NYY3	2.467e-19
	DMPK	Q09013	1.11e-15
	RIOK3	O14730	2.22e-15
	TBK1	Q9UHD2	3.22e-15
	GRK7	Q8WTQ7	5.396e-14
	CAMKK2	Q96RR4	5.407e-14
	DCLK1	O15075	6.017e-14
	DCLK2	Q8N568	9.337e-14
	STK16	O75716	2.047e-13
	CASK	O14936	2.802e-13
	DAPK2	Q9UIK4	3.785e-13
	COQ8A	Q8NI60	8.624e-13
	DAPK1	P53355	1.179e-12
	STK10	O94804	1.498e-12
	HIPK3	Q9H422	1.998e-12
	TNK1	Q13470	2.219e-12
	MAPK15	Q8TD08	3.015e-12
	DCLK3	Q9C098	1.095e-11
	PLK3	Q9H4B4	1.168e-11
	COQ8B	Q96D53	1.185e-11
	HIPK1	Q86Z02	1.308e-11
	MAP3K1	Q13233	1.333e-11
	CAMKK1	Q8N5S9	1.398e-11
	WEE2	P0C1S8	1.693e-11
	EPHB3	P54753	2.147e-11
	RIOK1	Q9BRS2	3.303e-11
	CDK15	Q96Q40	3.696e-11
	ROS1	P08922	4.618e-11
GSK1379763	PTK2	Q05397	4.446e-98
	INSR	P06213	1.216e-58
	TBK1	Q9UHD2	4.823e-58
	SYK	P43405	1.92e-53
	KDR	P35968	2.235e-38
	ERBB2	P04626	7.395e-37
	PDGFRA	P16234	6.12e-35
	PDGFRB	P09619	1.648e-33
	MAPK8	P45983	8.435e-29
	LCK	P06239	3.774e-22
	MAPK9	P45984	5.033e-19
	SRC	P12931	5.848e-18
	AURKA	O14965	1.585e-17
	PLK1	P53350	1.11e-16
	ABL1	P00519	4.441e-16
	MET	P08581	1.443e-15
	AURKB	Q96GD4	5.44e-15
	LIMK2	P53671	7.361e-14
	CDK4	P11802	9.859e-14
	FLT4	P35916	1.66e-13
	TNK2	Q07912	4.642e-13
	KIT	P10721	4.747e-13
	TEK	Q02763	6.809e-13
	RET	P07949	7.421e-13
	JAK3	P52333	1.852e-12
	TAOK1	Q7L7X3	1.988e-12
	TTK	P33981	3.24e-12
	GSK3B	P49841	1.124e-11
	ZAK	Q9NYL2	6.169e-11
	FLT3	P36888	6.522e-11
	GSK3A	P49840	6.975e-11

Supplementary References

1. Cichońska A. *et al.* Crowdsourced mapping of unexplored target space of kinase inhibitors. Zenodo 2021, doi:10.5281/zenodo.4648011.

2. Davis M.I. *et al.* Comprehensive analysis of kinase inhibitor selectivity. Nature Biotechnology 2011, 29(11):1046-1051.

3. Fabian M.A. *et al.* A small molecule-kinase interaction map for clinical kinase inhibitors. Nature Biotechnology 2005, 23(3):329-336.

4. Drewry D.H. *et al.* Progress towards a public chemogenomic set for protein kinases and a call for contributions. PloS One 2017, 12(8):e0181585.
