# Supplementary material for: Crowdsourced mapping of unexplored target space of kinase inhibitors
Source: Nat Commun. 2021 Jun 3;12:3307. doi: 10.1038/s41467-021-23165-1 (PMC8175708; doi:10.1038/s41467-021-23165-1)
Supplement: Supplementary file 3 — Description of Additional Supplementary Files [file 41467_2021_23165_MOESM3_ESM.docx]

File Name: Supplementary Data 1.xlsx

Description: Challenge Round 1 and Round 2 pK_d_ and %Inhibition data.

File Name: Supplementary Data 2.xlsx

Description: Additional compound-target pairs selected for DiscoverX pK_d_ profiling.
